# The NF-κB-dependent and -independent transcriptome and chromatin landscapes of human coronavirus 229E-infected cells

**DOI:** 10.1371/journal.ppat.1006286

**Published:** 2017-03-29

**Authors:** Michael Poppe, Sascha Wittig, Liane Jurida, Marek Bartkuhn, Jochen Wilhelm, Helmut Müller, Knut Beuerlein, Nadja Karl, Sabin Bhuju, John Ziebuhr, M. Lienhard Schmitz, Michael Kracht

**Affiliations:** 1 Rudolf Buchheim Institute of Pharmacology, Justus Liebig University Giessen, Giessen, Germany; 2 Institute for Genetics, Justus Liebig University Giessen, Giessen, Germany; 3 Universities of Giessen and Marburg Lung Center (UGMLC), Justus Liebig University Giessen, Giessen, Germany; 4 Institute of Medical Virology, Justus Liebig University Giessen, Giessen, Germany; 5 Helmholtz Centre for Infection Research, Braunschweig, Germany; 6 Institute of Biochemistry, Justus Liebig University Giessen, Giessen, Germany; Centro Nacional de Biotecnología (CNB, CSIC), SPAIN

## Abstract

Coronavirus replication takes place in the host cell cytoplasm and triggers inflammatory gene expression by poorly characterized mechanisms. To obtain more insight into the signals and molecular events that coordinate global host responses in the nucleus of coronavirus-infected cells, first, transcriptome dynamics was studied in human coronavirus 229E (HCoV-229E)-infected A549 and HuH7 cells, respectively, revealing a core signature of upregulated genes in these cells. Compared to treatment with the prototypical inflammatory cytokine interleukin(IL)-1, HCoV-229E replication was found to attenuate the inducible activity of the transcription factor (TF) NF-κB and to restrict the nuclear concentration of NF-κB subunits by (i) an unusual mechanism involving partial degradation of IKKβ, NEMO and IκBα and (ii) upregulation of TNFAIP3 (A20), although constitutive IKK activity and basal TNFAIP3 expression levels were shown to be required for efficient virus replication. Second, we characterized actively transcribed genomic regions and enhancers in HCoV-229E-infected cells and systematically correlated the genome-wide gene expression changes with the recruitment of Ser5-phosphorylated RNA polymerase II and prototypical histone modifications (H3K9ac, H3K36ac, H4K5ac, H3K27ac, H3K4me1). The data revealed that, in HCoV-infected (but not IL-1-treated) cells, an extensive set of genes was activated without inducible p65 NF-κB being recruited. Furthermore, both HCoV-229E replication and IL-1 were shown to upregulate a small set of genes encoding immunomodulatory factors that bind p65 at promoters and require IKKβ activity and p65 for expression. Also, HCoV-229E and IL-1 activated a common set of 440 p65-bound enhancers that differed from another 992 HCoV-229E-specific enhancer regions by distinct TF-binding motif combinations. Taken together, the study shows that cytoplasmic RNA viruses fine-tune NF-κB signaling at multiple levels and profoundly reprogram the host cellular chromatin landscape, thereby orchestrating the timely coordinated expression of genes involved in multiple signaling, immunoregulatory and metabolic processes.

## Author summary

Coronaviruses are major human and animal pathogens. They belong to a family of plus-strand RNA viruses that have extremely large genomes and encode a variety of proteins involved in virus-host interactions. The four common coronaviruses (HCoV-229E, NL63, OC43, HKU1) cause mainly upper respiratory tract infections, while zoonotic coronaviruses (SARS-CoV and MERS-CoV) cause severe lung disease, including acute respiratory distress syndrome (ARDS). The molecular basis for this fundamentally different pathology is incompletely understood. Our study provides a genome-wide investigation of epigenetic changes occurring in response to HCoV-229E. We identify at high resolution a large number of regulatory regions in the genome of infected cells that coordinate *de novo* gene transcription. Many of these genes have immunomodulatory functions and, most likely, contribute to limiting viral replication, while other factors may promote viral replication. The study provides an intriguing example of a virus that completes its entire life cycle in the cytoplasm while sending multiple signals to the nuclear chromatin compartment to adjust the host cell repertoire of transcribed genes. The approach taken in this study is expected to provide a suitable framework for future studies aimed at dissecting and comparing host responses to representative coronaviruses with different pathogenic potential in humans.

## Introduction

CoV infect a wide range of host species and cell types, but only six CoV are known to cause disease in humans [1]. Four human coronaviruses, HCoV‑229E, HCoV‑OC43, HCoV‑NL63 and HCoV‑HKU1, are mainly associated with upper respiratory infections, while severe acute respiratory syndrome (SARS)-CoV and middle east respiratory syndrome (MERS)-CoV may cause serious pathology in the lower respiratory tract, with acute lung injury, respiratory failure and death occurring in a significant number of infected individuals [1]. Lung biopsies obtained from SARS patients or infected primates consistently showed increased expression levels of (i) proinflammatory cytokines, such as interleukin(IL)-1, TNF and IL-6, (ii) chemokines, such as IL-8 (CXCL8), IP-10 (CXCL10) and MCP-1 (CCL2) and (iii) several other NF-κB target genes, suggesting that CoV infection triggers prototypical innate immune reactions involving the upregulation of inflammatory genes [2–4]. Other reports revealed a more diverse and incoherent pattern of host cell gene expression in response to SARS-CoV, MERS-CoV and HCoV-229E, respectively [5–7]. To date, it remains unclear if these differences are a consequence of different CoV strains/isolates and cell types being used in the respective studies or result (at least in part) from additional confounding factors, such as paracrine effects of cytokines or chemokines secreted during infection. Studies that define (i) the gene sets that are expressed specifically in CoV-infected but not bystander cells and (ii) the signaling pathways triggered in response to viral replication in these infected cells are still lacking [8]. Also, it remains to be studied if changes in host cellular mRNA levels in infected cells result from differential transcription rates of specific genes, altered mRNA stability, or both mechanisms.

Transcriptional gene regulation in response to pathogens involves the activation of a cascade of signal-regulated events that trigger *de novo* recruitment of RNA polymerase II or the release of paused RNA pol II at transcriptional start sites (TSS) [9]. Ongoing transcription requires phosphorylation of RNA pol II at the C-terminal domain (CTD) heptapeptide repeats by CTD kinases. This modification marks the transition from the preinitiation to the initiation complex and is also characteristic for pausing of catalytically active pol II 40–60 nucleotides downstream from the TSS [10]. Accordingly, P(S5)-pol II levels are high at the 5’ end of transcribed genes and decrease in a gene-specific manner towards the 3’ end during productive elongation within each cycle of pol II-mediated mRNA synthesis [11]. A potent signal-dependent TF that regulates transcription in innate immunity is NF-κB, a (hetero)dimer composed of the subunits p65, p105/p50 or p100/p52, c-Rel or RelB [12]. These subunits are retained in the cytoplasm by inhibitor of NF-κB (IκB) proteins. In the canonical NF-κB pathway, phosphorylation by IκB kinases (IKKs) and subsequent ubiquitylation and proteasomal degradation of IκBs liberates p65 subunits. In the non-canonical pathways, p100 or p105 are proteolytically processed to generate free p52 or p50, respectively [13]. Liberated NF-κB subunits then translocate to the nucleus, where their DNA binding and activity is modulated in a cell type- and stimulus-specific manner, a complex process that is mainly controlled by the chromatin accessibility of NF-κB motifs, post-translational modifications and cooperative interaction(s) with other TFs or coregulators such as histone acetyltransferases (HATs) [14].

Transcriptional processes often depend on a highly abundant array of gene-regulatory elements in the human genome, called enhancers [15]. Within enhancer regions, histone H3K4me1 and H3K27ac are strongly associated with combinations of *cis*-elements that primarily encode binding motifs for lineage-determining TFs, but some enhancers also recruit stimulus-specific inducible TFs [16]. Active enhancers can be distinguished by the presence of regulated histone acetylation at H3K27. Information on possible cooperative activities of TFs in enhancer-dependent gene regulatory networks may be inferred from the motif architecture of the underlying *cis*-elements [15, 17]. While enhancers are known to represent binding platforms for multiple TFs, the specific relationships between cooperative TF binding, enhancer selection and subsequent patterns of gene regulation in response to external cues including viral infections are still not very well understood [14, 16].

In the current study, we investigated, in a systematic manner, the dynamic gene expression profile in cells infected with HCoV-229E and compared these changes to those induced by the major inflammatory cytokine IL-1. We also assessed the chromatin status of HCoV-229E-infected cells at a genome-wide level. Our data suggest that cytoplasmic RNA viruses may exert profound effects on cellular chromatin structure and gene expression, involving attenuated nuclear regulation of the NF-κB pathway and activation of an extensive set of genomic enhancers.

## Results

### Identification of differentially regulated host cell genes in response to coronavirus replication

**Fig 1:**
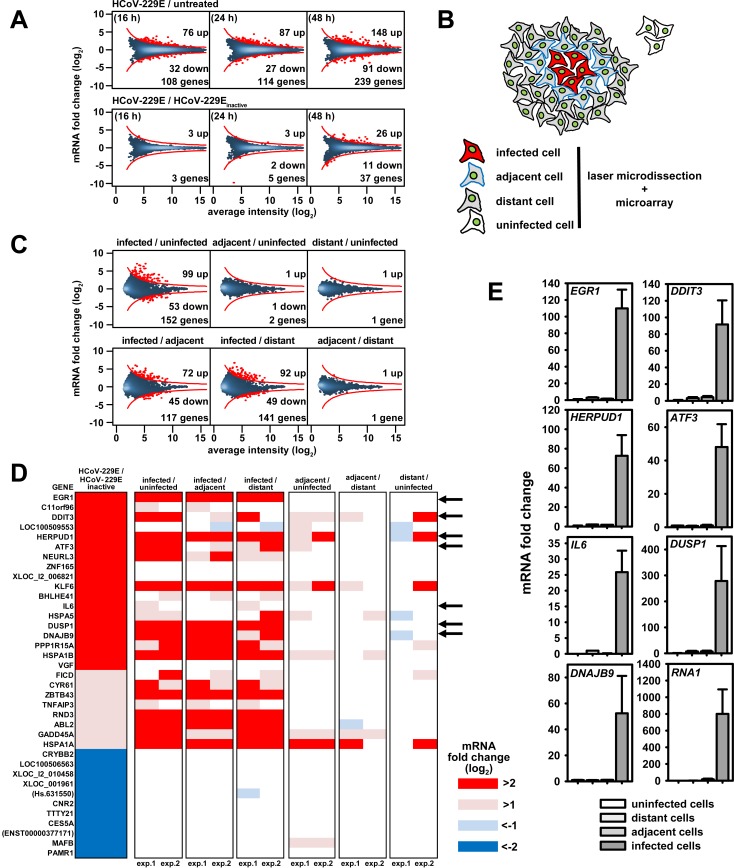
Identification of host cell genes directly regulated in response to HCoV-229E replication in human lung epithelial cells. (A) A549 lung epithelial cells were infected with HCoV-229E and collected for further analyses at 16, 24 and 48 h p.i.. As controls, cells were mock infected or incubated with heat-inactivated virus and collected at the time points indicated above. Transcriptomes for the respective cells were determined using Agilent microarrays. Graphs represent MA plot visualizations of changes in gene expression (M, log_2_ ratios) versus mRNA abundance (A, log_2_ average fluorescence intensity). An intensity-based cut off was calculated and red dots above or below the red lines represent genes with differential expression in response to virus. (B) Scheme of cell populations isolated from HCoV-229E-infected A549 cells at 48 h p.i. or from uninfected cells by laser microdissection. (C) Two independent series of microarray experiments were performed from laser microdissected cells and data were averaged. MA plots showing transcriptome changes in cells infected with HCoV-229E compared to adjacent or distant cells or to noninfected cells collected from a separate mock-infected culture. D) The 37 genes that were specifically regulated by HCoV-229E as shown in (A), lower right panel, were selected and their expression in both independent experiments of laser microdissected cell populations as determined in (C) was visualized by heatmaps. Arrows indicate seven genes with differential expression levels that were further validated by RT-qPCR as shown in (E). Bar graphs displayed in (E) represent mean changes +/- s.e.m. from 3 independent experiments. RNA1 represents the HCoV-229E genome RNA as determined using replicase gene (nsp8)-specific primers. See also S1 and S2 Figs and S1 and S2 Tables.

To analyze HCoV-229E-induced host cell gene responses in a systematic manner, we first conducted a series of transcriptome-wide studies using the A549 lung epithelial carcinoma cell model. To detect transcriptional changes caused by viral replication, the cells were either infected at low multiplicity with (replication-competent) HCoV-229E (MOI 0.001) or inoculated with replication-incompetent (heat- or UV-inactivated) HCoV-229E. At 16 and 48 h post infection (p.i.), increasing amounts of HCoV-229E RNA (as assessed by RT-PCR using primer pairs specific for the nsp8 and spike protein coding regions, respectively) were detectable in HCoV-229E-infected cells, but not in control cells inoculated with heat- or UV-inactivated HCoV-229E (S1A Fig). To determine the impact of HCoV-229E infection on the entire host cell transcriptome, we used Agilent microarrays containing 60,000 probes covering annotated genes and non-coding RNAs. Infection with HCoV-229E was found to cause differential expression of 108 genes at 16 h p.i. and 239 genes at 48 h p.i. (Fig 1A, upper panels and S1 Table). A nearly identical set of differentially expressed genes was identified in control cells inoculated with heat-inactivated virus, that is, in the absence of (detectable) viral replication (Fig 1A, lower left and middle panels), suggesting that the observed differential gene expression was caused to a significant extent by soluble factors present in the viral stock or interactions between cells and non-infectious virus particles. However, 2 days after infection we were also able to identify a specific set of 26 upregulated and 11 downregulated genes that were differentially regulated only in cells infected with replication-competent HCoV-229E but not in cells inoculated with inactivated virus (Fig 1A, lower right panel).

In a next set of experiments, we used immunofluorescence analysis of HCoV-229E nucleocapsid (N) protein expression to (i) monitor HCoV-229E infection and spread in A549 cells (S1B Fig) and (ii) characterize at the single-cell level the gene expression changes in infected as opposed to adjacent non-infected cells present in the same or separate culture(s). Laser microdissection coupled with immunofluorescence was used to excise (i) HCoV-229E-infected cells, (ii) cells immediately adjacent to infected cells, (iii) cells located at least 150 μm away from infected cells ("distant cells") and (iv) cells from a separate, non-infected cell culture (for details, see Fig 1B and S1B Fig). Two independent series of microarray studies revealed almost no changes of gene expression in adjacent, distant and uninfected cell populations, while infected cells showed a total of 99 up- and 53 down-regulated genes, respectively (Fig 1C and S2 Table). Overrepresentation (ORA) and gene set enrichment analyses (GSEA) for the genes identified in the time-course experiment (Fig 1A) and the laser-microdissected samples (Fig 1B and 1C) across all pair-wise comparisons identified the KEGG pathways 04141 (protein processing in the endoplasmic reticulum) and 03013 (RNA transport) as the pathways that were most significantly affected by HCoV-229E infection (S2A Fig). Visualization of the mRNA expression values in KEGG 04141 and 03013 pathway maps illustrates that many mRNAs are induced or suppressed at several crucial nodes within these pathways (S2B Fig). The initially identified set of 37 genes regulated by replicating virus only (Fig 1A) contained several strongly upregulated genes of these pathways and included transcription factors (*EGR1*, *DDIT3* (also called *CHOP*, *GADD153* or *C/EBPzeta*), *ATF3*, *KLF6*, *ZNF165*, *BHLHE41*, *ZBTB43*), cytokines and stress signaling proteins (*IL6*, *GADD45A*/*DDIT1*, *TNFAIP3/A20*), phosphatases (*DUSP1*), and further factors involved in ER stress (*DNAJB9/ERDJ4*, *NEURL3*, *HERPUD1*, *HSPA5/GRP78/BIP*) or regulators of translation (*PPP1R15A/GADD34*, *HSPA1A/HSP701A*, *HSPA1B/HSP701B*). Fig 1D summarizes the observed changes in the expression levels of these genes and shows that the differential expression is specific for (and restricted to) infected cell populations. The upregulation of selected genes was confirmed by independent RT-qPCR experiments (Fig 1E). Taken together, these data suggest that HCoV-229E replication regulates complex sets of genes involved in transcription, stress responses, cytokine signaling and nucleotide metabolism.

### Comparison of HCoV-229E- and IL-1-regulated transcriptomes

**Fig 2:**
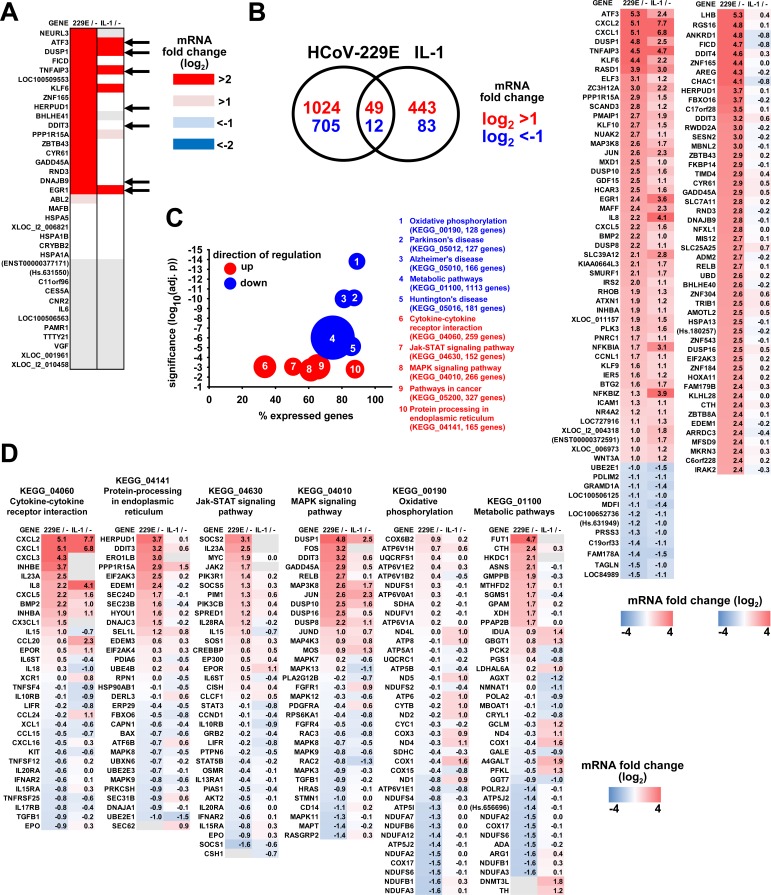
Comparison of HCoV-229E- and IL-1-regulated transcriptomes reveals common and coronavirus-specific sets of genes belonging to specific biological pathways. HuH7 cells were infected with HCoV-229E for 24 h or were left uninfected. In parallel, cell cultures were treated with IL-1 for 1 h. Transcriptome analyses were performed from total RNA using Agilent microarrays. Data from four independent experiments were pooled of which the last two included IL-1 stimulation. (A) Shown is the overlap of HCoV-229E-regulated genes in HuH7 cells with the 37 genes regulated in A549 cells as identified in Fig 1. Black arrows indicate seven genes whose expression changes were validated by RT-qPCR as shown in S3A Fig. (B) Venn diagram of all genes regulated more than 2-fold that were significantly expressed over background in response to HCoV-229E or IL-1. The left heatmap shows the averaged ratios of the 61 genes that were regulated by both HCoV-229E and IL-1. The right heatmap shows the top 50 genes that were regulated by HCoV-229E only. Individual gene expression ratios for the CoV-specific regulated genes are shown in S3B Fig. (C) Gene set enrichment analyses were used to identify HCoV-229E-regulated KEGG pathways from the four independent experiments. Shown are the ten most strongly down- (blue colors) or upregulated (red colors) pathways as indicated by the adjusted p values. Sizes of bubbles correspond to the total number of genes annotated to each KEGG pathway which varied from 127 genes (KEGG_05012) up to 1113 genes (KEGG_01100). The x-axis indicates the relative number of genes of each pathway that were found to be expressed in HuH7 cells. D) Relative expression data for the top ten up- and downregulated genes of the indicated pathways in response to virus infection or IL-1 treatment were selected. Gene lists were merged and heatmaps show ratios of expression and provide a comparison of virus-infected cells with IL-1 treated cells. Gray colors in (A) and (D) show genes with no detectable expression in a particular condition. See also S3 and S4 Figs and S3 Table.

While A549 cells are a suitable model for studying pathology caused by HCoV-229E in lung epithelial cells, their low infection efficiency with HCoV-229E precluded in-depth molecular analyses requiring very large numbers of infected cells. We, therefore, decided to use HuH7 cells, a widely used human hepatoma cell line that is known to support efficient HCoV-229E replication and infectious particle formation, for subsequent high-resolution analyses at the genome-wide level [18]. As a control, we treated HuH7 cells with IL-1, which allowed us to compare HCoV-229E-linked gene signatures with those of a well characterized cytokine that regulates a broad range of inflammatory genes through stress kinase or NF-κB pathways. HuH7 cells were either infected with HCoV-229E (MOI = 1) or were treated with IL-1 for 1 h and subsequently used for transcriptome analyses. We first asked if the HCoV-229E-linked gene signature observed in A549 cells could also be confirmed in HuH7 cells. We found that 19 out of 26 genes upregulated by HCoV-229E in A549 cells were also upregulated in HuH7 cells, defining a cell type-independent set of virus-induced host cell target genes (Fig 2A). The genes downregulated in A549 cells were not expressed in HuH7 cells (Fig 2A). The kinetic regulation of seven of these genes and their specific induction in cells productively infected with HCoV-229E but not in cells incubated with heat-inactivated virus was validated by RT-qPCR (S3A Fig). IL-1 induced six of these genes (*ATF3*, *DUSP1*, *TNFAIP3*, *KLF6*, *PPP1R15A*, and *EGR1*) indicating that HCoV-229E shares pathways with this cytokine but activates additional specific sets of genes (Fig 2A). At the genome-wide level, HCoV-229E was found to upregulate 1,073 genes and to downregulate 717 genes, whereas IL-1 induced 492 genes and downregulated 95 genes by at least 2-fold on average in repeated microarray studies (Fig 2B, Venn diagrams). The left heatmap in Fig 2B shows all 61 jointly regulated genes which includes well-characterized inflammatory genes (e.g. *IL8*, *CXCL1*, *CXCL2*, *CXCL5*, *CXCL6*, *ICAM1*) as well as signaling proteins regulated during inflammatory cell activation (e.g. *DUSP1*, *DUSP8*, *DUSP10*, *EGR1*, *c-JUN*, *NFKBIA*, *NFKBIAZ*, *TNFAIP3*, *MAP3K8 (TPL-2/COT)*, *ZC3H12A (MCPIP1*)). Despite these similarities we observed a substantial number of genes which are upregulated by HCoV-229E, but not by IL-1. The right heatmap in Fig 2B shows the top 50 genes that are specifically upregulated by HCoV-229E. These genes were consistently induced by HCoV-229E in all four individual microarray experiments and were not regulated by heat-inactivated virus (S3B Fig). The differential regulation patterns in response to IL-1 or HCoV-229E were further corroborated by analyzing mRNA expression of representative genes by RT-qPCR (S4A Fig). These data clearly show the major impact of HCoV-229E replication on inflammatory pathways but also reveal much broader virus-induced effects on cellular gene expression. To determine if HCoV-229E-induced genes are enriched for specific biological pathways, the complete microarray data sets were analyzed by GSEA. Fig 2C shows the top ten pathways with significant enrichment of regulated genes and the overall direction of regulation of the entire gene sets. The complete list of expression values and the components for all KEGG pathways of Fig 2C are shown in S3 Table. Fig 2D highlights the similarities and differences of HCoV-229E- versus IL-1-regulated changes in mRNA expression of individual genes in six of these pathways, all of which representing intracellular processes. In four KEGG pathways (04060, 04141, 04630, 04010), HCoV-229E replication mainly caused an upregulation of genes (e.g. *CXCL2*, *HERPUD1*, *DUSP1*, *FUT1*), while in pathway 00190 the virus infection was mainly associated with a downregulation of genes. In comparison, and consistent with its role as a MAPK pathway-activating cytokine, IL-1 was found to primarily upregulate genes in the pathways 04060 (cytokine-cytokine receptor interaction) and 04010 (MAPK signaling pathway). Together, the data shown in Fig 1 and Fig 2 define a core set of genes that are consistently induced by HCoV-229E in both A549 and HuH7 cells. Most of these genes are specifically regulated by HCoV-229E, while another subset of genes is shared with IL-1.

### Chromatin status and transcriptional regulation of HCoV-229E-upregulated genes

**Fig 3:**
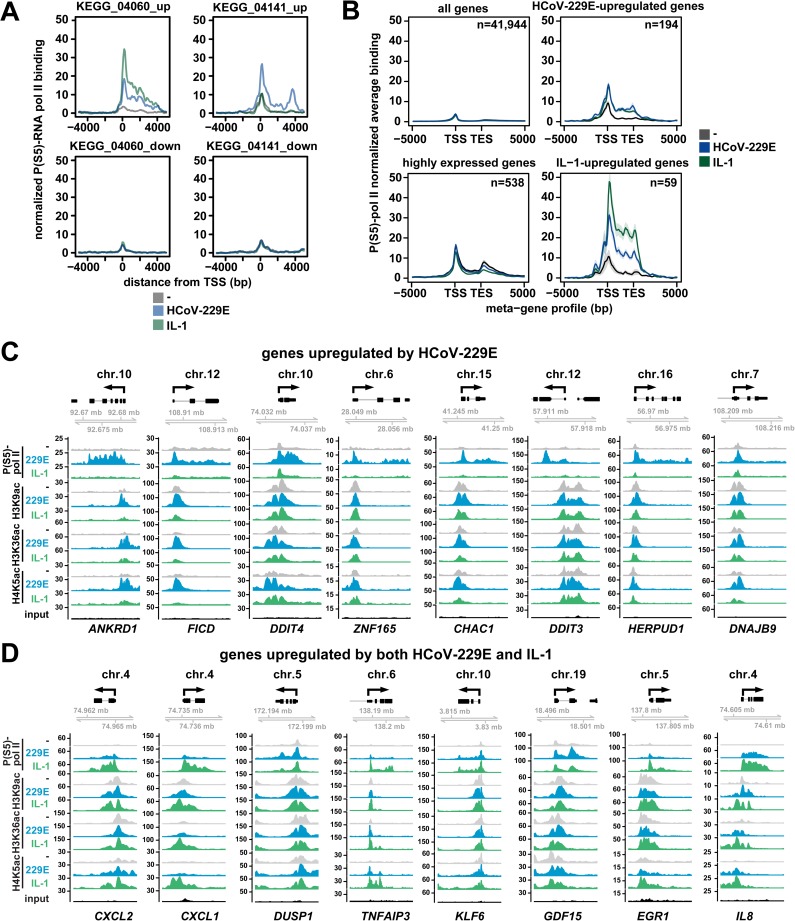
HCoV-229E replication is linked to specific binding patterns of active RNA polymerase II that correlate with mRNA expression and inducible acetylation of histone H4K5. (A, B) HuH7 cells were infected with HCoV-229E for 24 h or were treated with IL-1 for 1 h or were left untreated. ChIP-seq experiments were performed using an antibody against the transcription-initiating serine 5-phosphorylated form of RNA polymerase II (P(S5)-pol II). Mapped reads were centered at the transcriptional start sites (TSS) of up- or downregulated genes of KEGG clusters 04060 or 04141 as identified in Fig 2D and visualized as average profiles for each KEGG cluster. B) Meta-gene profiles of P(S5)-pol II recruitment according to mRNA expression levels for all genes, for highly expressed genes (log_2_(intensity) on microarrays >13) and for HCoV-229E- and IL-1-upregulated genes +/- 5 kb around gene bodies. Lines indicate means, shadows indicate confidence intervals (mean +/- 2 s.e.m.). Regulated genes were selected based on 4-fold cut-offs using microarray data from two (IL-1) or four (HCoV-229E) biological replicates as shown in Fig 2. (C, D) Browser views of P(S5)-pol II recruitment patterns and histone modifications of genes induced specifically by HCoV-229E (C) or by both IL-1 and HCoV-229E (D). Genes were selected on the basis of their mRNA induction as shown in Fig 2B and 2D. Y-axes show normalized read counts. See also S4 Fig.

To investigate if and to what extent HCoV-229E affects host cell mRNA expression at the transcriptional level, we analyzed the Ser5 phosphorylated form of RNA polymerase II (P(S5)-pol II) that accumulates during formation of the transcription initiation complex near the transcriptional start site (TSS) [11]. In order to investigate the recruitment of P(S5)-pol II to the promoter of the HCoV-inducible *IL8* gene, we performed ChIP experiments which showed a time-dependent recruitment with a maximum at 24 h p.i. of RNA polymerase II and its phosphorylated form to the promoter/TSS region of the *IL8* gene (S4B Fig). This condition was chosen for ChIP-seq experiments using the P(S5)-pol II antibody. The data were then used to assemble averaged profiles of P(S5)-pol II recruitment for groups of up- and downregulated genes that belong to the KEGG pathways and which were affected by HCoV-229E replication and/or IL-1 treatment. Upregulated genes belonging to the cytokine pathway KEGG_04060 showed increased recruitment of P(S5)-pol II by both, HCoV-229E and IL-1, and a prototypical decline of P(S5)-pol II towards the 3’ ends of the genes (Fig 3A). In this group of genes, IL-1-mediated P(S5)-pol II signals were stronger than the CoV-mediated effects (Fig 3A), in line with the stronger induction of mRNA expression of genes such as *IL8* or *CXCL2* (Fig 2D). Upregulated genes belonging to the ER stress pathway KEGG_04141 showed no regulation by IL-1, but strong upregulation of P(S5)-pol II signals by HCoV-229E (Fig 3A), again correlating with mRNA expression (Fig 2D). For downregulated genes of KEGG pathways 04060 or 04141, we found only low levels of constitutive and unregulated P(S5)-pol II occupancy (Fig 3A). This observation suggests that altered mRNA levels of these genes may be regulated by post-transcriptional mechanisms independently from a low level of basal transcription. A meta-gene analysis of P(S5)-pol II recruitment across the entire transcriptome showed a good correlation of P(S5)-pol II recruitment with mRNA expression. For 194 genes shown to be strongly (≥ 4-fold) upregulated by HCoV-229E and for 59 genes shown to be regulated by both IL-1 and HCoV-229E, an inducible P(S5)-pol II recruitment could be confirmed (Fig 3B). Collectively, these data indicate that HCoV-229E induces a genome-wide transcriptional response to upregulate a broad range of cellular genes.

To examine histone modifications at genomic regions with increased recruitment of RNA polymerase II in HCoV-229E-infected cells, we analyzed the acetylation patterns of H3K9, H3K36 and H4K5. All three modifications are highly enriched at promoters, TSS and gene bodies, where they function to open nucleosomal DNA and additionally provide binding sites for multiple coactivators [19]. Fig 3C shows the histone acetylation pattern for 8 genes whose transcription is specifically induced by HCoV-229E as indicated by the P(S5)-pol II recruitment pattern. At these genes, basal H3K9ac was found to be high and only regulated for two genes (*ANKRD1*, *FICD*). At all genes, the basal H3K36ac level was increased by HCoV-229E but not by IL-1. H4K5ac levels were low in uninfected and IL-1 treated cells, but were consistently increased by HCoV-229E. These profiles show that H4K5ac and, for most genes, H3K36ac are characteristic inducible histone marks of HCoV-229E-induced genes. Fig 3D shows examples of read count distribution for eight genes, which are induced by both HCoV-229E and IL-1. These genes show high levels of non-regulated H3K9ac at their promoters and five of them show inducible H3K36ac and H4K5ac (*CXCL2*, *CXCL1*, *TNFAIP3*, *EGR1*, *IL8*). As shown in Fig 2B (left heatmap), some genes are stronger induced at the mRNA level by IL-1 (e.g. *CXCL2*, *CXCL1*, *EGR1*, *IL8*), correlating well with the observed stronger P(S5)-pol II recruitment to these genes. Quantification of read counts confirmed similar inducible changes in histone modifications and P(S5)-pol II recruitment for the upregulated genes in KEGG pathway 04060 and KEGG pathway 04141, but not for the downregulated genes (S4C Fig). Taken together, the data show that HCoV-229E activates specific sets of host cell genes at the transcriptional level by increasing phosphorylation of RNA pol II and acetylation of H3K36 and H4K5 at the promoter and TSS regions.

### Modulation of NF-κB signaling by HCoV-229E and functional relevance of NF-κB regulators for HCoV-229E-induced target genes and viral replication

**Fig 4:**
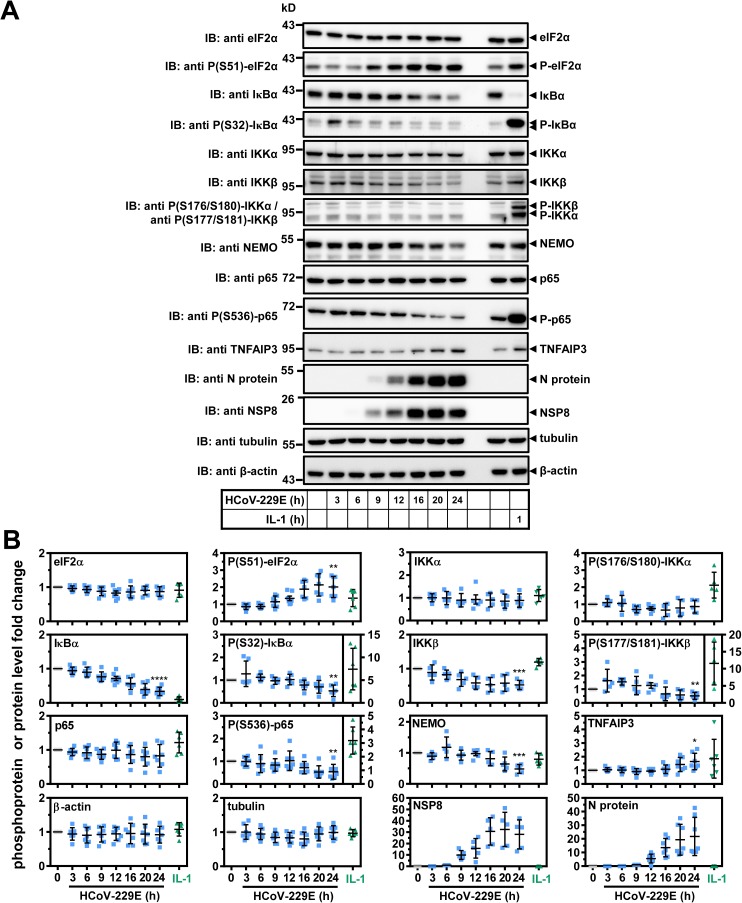
Differential activation or repression of regulators of NF-κB signaling by HCoV-229E or IL-1. (A) HuH7 cells were infected with HCoV-229E for the indicated times, or were treated with IL-1 for 1 h or were left untreated. Expression and phosphorylation (P) of the indicated proteins was analyzed by immunoblotting of whole cell extracts. Tubulin or β-actin antibodies were used to control for equal loading. (B) Graphs show quantification of relative protein/P-protein levels including means +/- s.d. from five to seven independent experiments. For viral proteins the 6 h (nsp8) or 9 h (N protein) protein levels were set as one. Asterisks indicate significance of differences as obtained from one-tailed t-tests comparing cells infected for 24 h with untreated cells (**** p<0.0001, *** p<0.001, ** p<0.005, * p<0.05). See also S5 Fig.

The gene set that was transcriptionally induced by both HCoV-229E and IL-1 contained several well-characterized NF-κB target genes, such as *IL8*, *CXCL2* and *TNFAIP3*. This raised the question of whether HCoV-229E elicits non-canonical NF-κB signaling or activates the canonical NF-κB pathway, the latter involving IκBα degradation [20]. To address this question, cells were infected with HCoV-229E for different periods and signaling events were analyzed by Western blot experiments in comparison to cells that were stimulated for 1 h with IL-1. This cytokine caused strong phosphorylation (mean fold 7.4 +/- 4.6 s.d.) and nearly complete degradation of IκBα (mean fold 0.10 +/- 0.073 s.d.) (Fig 4A and 4B). IκBα protein levels were also reduced at 24 h after infection with HCoV-229E but to a significantly lesser extent (mean fold 0.34 +/- 0.12 s.d.) and without a concomitant virus-mediated increase in IκBα phosphorylation at this time point (Fig 4A and 4B). A transient early induction of Ser32 phosphorylation of IκBα by HCoV-229E after 3 h of infection was only seen inconsistently in two out of six experiments (Fig 4A and 4B). IL-1 did not affect the expression of the core IKK complex catalytic subunits IKKα and IKKβ or of the regulatory subunit NEMO, but the cytokine strongly activated the phosphorylation of IKKβ (mean fold 12 +/- 6.3 s.d.) and moderately that of IKKα (mean fold 2.1 +/- 0.75 s.d.) (Fig 4A and 4B). In contrast, HCoV-229E infection led to decreased levels of P- IKKβ (mean fold 0.52 +/- 0.26 s.d.), IKKβ (mean fold 0.53 +/- 0.13 s.d.) and NEMO (mean fold 0.48 +/- 0.14 s.d.) but remained without effects on P-IKKα / IKKα (Fig 4A and 4B). Additionally, HCoV-229E was found to induce the expression of TNFAIP3 (A20) (mean fold 1.6 +/- 0.60 s.d.), a potent cytosolic inhibitor of the IKK complex (Fig 4A and 4B) [21].

**Fig 5:**
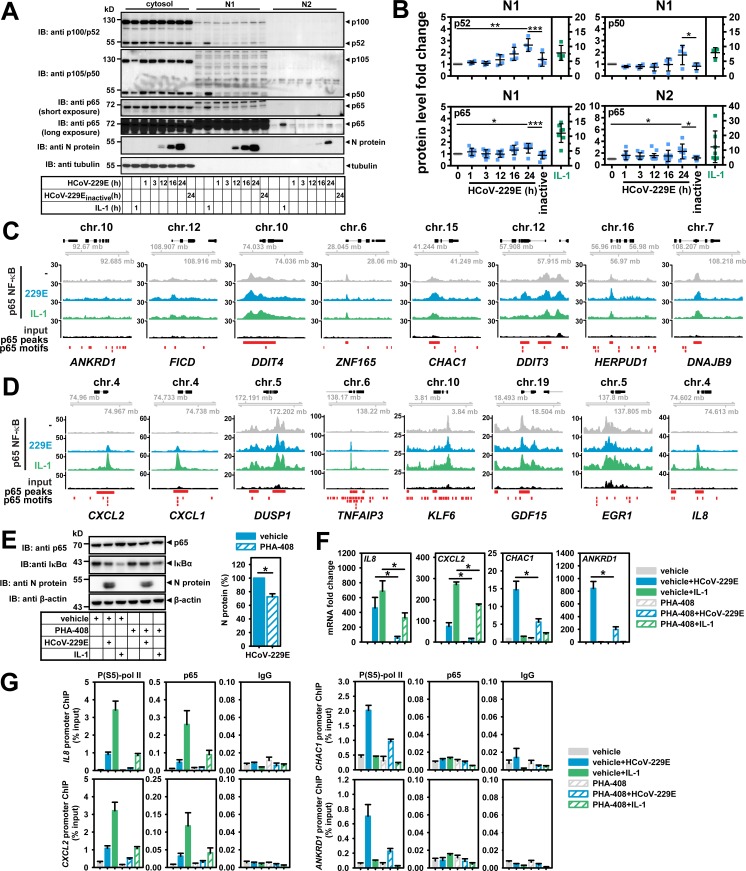
Chromatin recruitment of NF-κB subunits to promoters of IL-1 or HCoV-229E-regulated target genes. (A) HuH7 cells were infected with HCoV-229E or heat-inactivated HCoV-229E (inactive) for the indicated times, or were treated with IL-1 for 1 h or were left untreated. Then, cytosolic, soluble nuclear (N1) and insoluble nuclear chromatin (N2) fractions were prepared and equal amounts of proteins were analyzed for the expression and translocation of NF-κB subunits and viral nucleocapsid (N) protein. (B) Graphs show quantification of relative protein levels including means +/- s.d. from four independent experiments. p65 levels from three experiments were determined by two different antibodies resulting in a total of seven measurements. Asterisks indicate significance of differences as obtained from one-tailed t-tests comparing cells infected for 24 h with untreated cells, or intact virus versus heat-inactivated virus, respectively (*** p<0.0005, ** p<0.005, * p<0.05). (C, D) Cross-linked chromatin of uninfected, IL-1-treated or HCoV-229E-infected cells was prepared followed by p65 immunoprecipitation and deep sequencing of p65-bound DNA fragments. Depicted are p65 ChIP-seq browser profiles for eight genes whose mRNA levels are specifically regulated by HCoV-229E (C) and for eight genes whose mRNA levels are regulated by both HCoV-229E and IL-1 (D) as shown in Fig 2B. (E) HuH7 cells were treated with solvent (DMSO, vehicle) or with the IKKβ inhibitor PHA-408 (5 μM) for 30 min followed by infection with HCoV-229E for 24 h or treatment with IL-1 for 1 h. Equal amounts of proteins from whole cell extracts were analyzed for the expression of p65 NF-κB, IκBα and viral N protein. β-actin antibodies were used to control for equal loading. Right graph: Quantification of relative N protein levels (mean +/- s.e.m.) from four independent experiments. (F) Mean relative changes +/- s.e.m. of mRNA expression of HCoV-229E or IL-1 target genes from four independent experiments of cells treated as in (E). (G) Recruitment of phosphorylated RNA pol II and p65 to the promoters of these target genes. Shown are results (mean +/- s.e.m.) from two independent ChIP-PCR experiments, IgG precipitations served as negative controls. The asterisks indicate significance of differences as obtained from t-tests (* p<0.01). See also S5 and S6 Figs.

**Fig 6:**
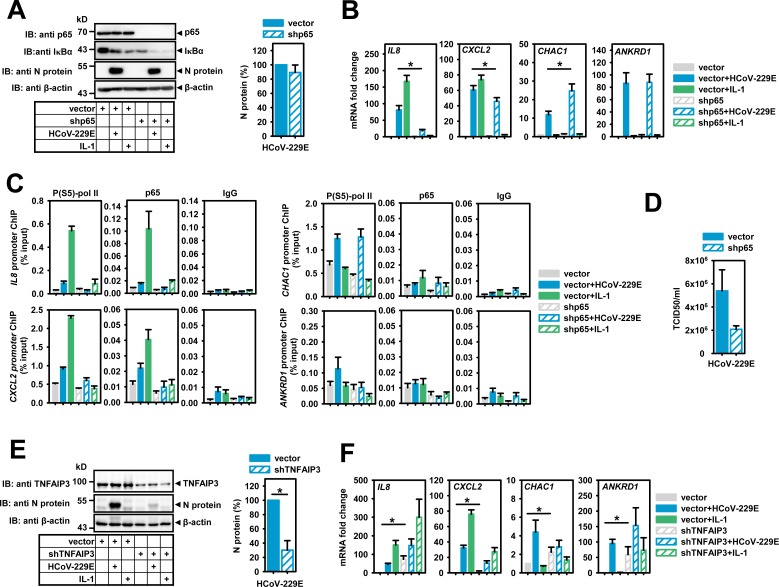
Suppression of p65 NF-κB or TNFAIP3 (A20) affect HCoV-229E replication and transcriptional regulation of NF-κB target genes. HeLa cells were transiently transfected with empty pSuper (vector), pSuper encoding shRNAs directed against p65 (B-D) or pLKO.1 encoding shRNAs against TNFAIP3 (E, F). One day later, cells were selected for 48 h (shp65) or 72 h (shTNFAIP3) in 1 μg/ml puromycin. Then, cells were infected with HCoV-229E for 24 h or were treated with IL-1 for 1 h. (A) Equal amounts of proteins from whole cell extracts were analyzed for the expression of p65 NF-κB, IκBα and viral N protein. β-actin antibodies were used to control for equal loading. Right graph: Quantification of relative N protein levels (mean +/- s.e.m.) from six independent experiments. (B) Mean relative changes +/- s.e.m. of mRNA expression of HCoV-229E or IL-1 target genes from six independent experiments of cells treated as in (A). (C) Recruitment of phosphorylated RNA pol II or p65 to the promoters of these target genes. Shown are results from two independent ChIP-PCR experiments, IgG precipitations served as negative controls. (D) Viral titers (mean TCID50/ml +/- s.e.m.) were determined in the supernatants of the same cells used for the experiments described in (C). (E) Equal amounts of proteins from whole cell extracts were analyzed for the expression of TNFAIP3 and viral N protein. β-actin antibodies were used to control for equal loading. Right graph: Quantification of relative N protein levels (mean +/- s.e.m.) from five independent experiments. (F) Mean relative changes +/- s.e.m. of mRNA expression of HCoV-229E or IL-1 target genes from five independent experiments. The asterisks indicate significance of differences as obtained from t-tests (* p<0.05). See also S6 Fig.

Another IKK substrate is S536 of p65 NF-κB [22]. This modification was reduced upon HCoV-229E infection (mean fold 0.54 +/- 0.33 s.d.) but triggered by IL-1 (mean fold 3.2 +/- 1.0 s.d.) (Fig 4A and 4B). Together, these data suggest that HCoV-229E replication on the one hand inefficiently triggers NF-κB activity by an unusual mechanism involving partial and selective IκBα degradation, but on the other hand dampens NF-κB activity by leading to reduced expression of IKK core complex subunits. This negative regulation occurred at the translational level, as the mRNAs encoding the three IKK subunits and IκBα were weakly induced by virus (S5A Fig). Besides induction of TNFAIP3, these effects may involve (negative) regulatory steps at the level of translation, as HCoV-229E upregulated the phosphorylation of the eukaryotic translation initiation factor subunit eIF2α at S51 (mean 2.0 +/- 0.62 s.d.), corroborating data obtained previously for the avian infectious bronchitis virus (IBV) (Fig 4A and 4B) [23]. Next, we analyzed the amounts of NF-κB subunits in soluble (N1) and chromatin (N2) nuclear fractions in response to HCoV-229E infection or IL-1. Consistent with previously published data, IL-1 strongly increased the concentrations of p50, p52, p65 and c-Rel NF-κB subunits by 5 to 10-fold in the soluble N1 fraction, whereas p65 was the most prominently increased subunit stably associated with the N2 chromatin fraction (Fig 5A and 5B, S6A Fig) [24, 25]. In HCoV-229E-infected cells, a modest 1.5 to 3-fold increase of p65 was observed in the N1 fractions, but also of p50 and p52 which are characteristic for the activation of the non-canonical pathway (Fig 5A and 5B). This experiment also showed that the p65 protein was the only subunit that was detectably increased in the N2 chromatin fraction after virus infection. These small changes were significantly different from basal nuclear levels of NF-κB subunits and were not seen when inactive virus was used (Fig 5A and 5B). We also detected the HCoV-229E N protein in both nuclear compartments, confirming earlier studies on a partial nuclear localization of CoV N proteins (Fig 5A) (reviewed in [26]). Collectively, the data shown in Fig 4, Fig 5A and 5B and S6A Fig demonstrate that HCoV-229E infection causes an attenuated nuclear NF-κB response involving both the non-canonical and canonical NF-κB pathways. To answer the question of whether HCoV-229E selectively regulates nuclear functions of the N2 fraction-associated p65 NF-κB, we analyzed the chromatin recruitment of p65 NF-κB to distinct genomic loci by ChIP-seq experiments, focusing on the promoters of sixteen HCoV-229E- or IL-1-regulated genes for which virus-mediated changes in P(S5)-pol II occupancy and histone acetylation pattern had been identified in the experiments reported above (see Fig 3C and 3D). For genes that were activated by HCoV-229E but not by IL-1, we only observed relatively low and unregulated p65 signals (Fig 5C). In contrast, IL-1 strongly stimulated the recruitment of p65 to 5 of the 8 genomic regions that contained genes shown to be regulated at the mRNA and transcriptional level by both HCoV-229E and IL-1 (see above and Fig 5D) whereas, for HCoV-229E-infected cells, only a moderate increase of p65 binding to the *CXCL2*, *CXCL1*, *TNFAIP3* and *IL8* loci could be detected (Fig 5D). The latter pattern of p65-dependent transcriptional regulation was also seen for the *NFKBIA* gene which encodes the highly regulated IκBα protein (S5A–S5C Fig). The functional relevance of the NF-κB system for the transcriptional regulation of virus-induced (*CHAC1*, *ANKRD1*) versus virus- and cytokine-induced genes (*IL8*, *CXCL2*) was tested using the IKKβ inhibitor PHA-408 [27]. This compound partially blocked IL-1- and HCoV-229E-induced IκBα degradation in HuH7 cells (Fig 5E), suggesting that IKK activation also contributes to HCoV-229E-mediated NF-κB activation. Accordingly, PHA-408 also inhibited the virus-inducible mRNA expression of all 4 genes as well as the upregulation of *IL8* and *CXCL2* mRNAs by IL-1 (Fig 5F). ChIP-PCR experiments confirmed the virus- versus IL-1-specific p65 and P(S5)-pol II recruitment patterns presented above (Fig 5G). PHA-408 suppressed P(S5)-pol II recruitment to the *IL8*, *CXCL2*, *CHAC1* and *ANKRD1* loci and virus- or IL-1-inducible p65 recruitment to the *IL8* and *CXCL2* promoters (Fig 5G). The IKKβ inhibitor also partially suppressed viral replication by about 30% as assessed by measuring N protein levels (Fig 5E). This result is in line with a positive role of IKKs for viral replication that may proceed through substrates unrelated to NF-κB as shown for a few other RNA viruses [20]. However, the inhibitory effects of PHA-408 at the mRNA and chromatin levels were stronger than the suppression of viral protein synthesis, suggesting that the IKK-NF-κB pathway may also exert pro-viral functions (Fig 5E–5G). To address the function of NF-κB by a complementary experimental approach, p65 expression was downregulated by RNAi in HeLa cells, which unlike HuH7 cells are suitable for both efficient HCoV-229E infection (shown in S6B and S6C Fig) and transfection of shRNAs. Almost complete reduction of p65 suppressed steady-state protein levels of the p65 target gene IκBα but did not affect the IL-1- or virus-mediated IκBα degradation or viral N protein synthesis (Fig 6A). Knockdown of p65 suppressed virus- or IL-1-induced mRNA expression as well as P(S5)-pol II and p65 recruitment to the NF-κB target genes *IL8* and *CXCL2* (Fig 6B and 6C). Moreover, decreased viral titers in these cells suggested that p65 is required for a step subsequent to viral protein synthesis (Fig 6D).

Further evidence for a fundamental role of the IKK-NF-κB system in the HCoV-229E host response was derived from TNFAIP3 knockdown experiments performed under identical conditions. Reduction of TNFAIP3 protein resulted in a 70% inhibition of the infection rate as assessed by N protein levels (Fig 6E). At the same time, basal mRNA expression levels of *IL8* and *ANKRD1* were strongly increased (Fig 6F). As a result, the fold regulation of these genes by HCoV-229E or IL-1 was almost completely lost (Fig 6F). Basal *CXCL2* and *CHAC1* levels were less affected by TNFAIP3 knockdown, however, signal-mediated regulation by HCoV-229E or IL-1 was also inhibited (Fig 6F). Collectively, the data shown in Figs 5 and 6 suggest important functional roles for IKKβ, TNFAIP3 and p65 in virus-mediated changes of host cell gene expression and viral replication and reveal that HCoV-229E infection also restricts the maximal activity of the IKK complex. This occurs by inversely balancing the levels of positive regulators of the NF-κB pathway (IKKβ, NEMO) versus negative cytosolic regulators of the IKK complex (TNFAIP3, IκBα). This fine-tuning allows for some basal NF-κB activity that supports viral replication but limits high nuclear concentration of NF-κB subunits avoiding excessive transcription of inflammatory and other potentially antiviral host cell genes.

### Genome-wide recruitment of p65 NF-κB in the HCoV-229E-induced host response

**Fig 7:**
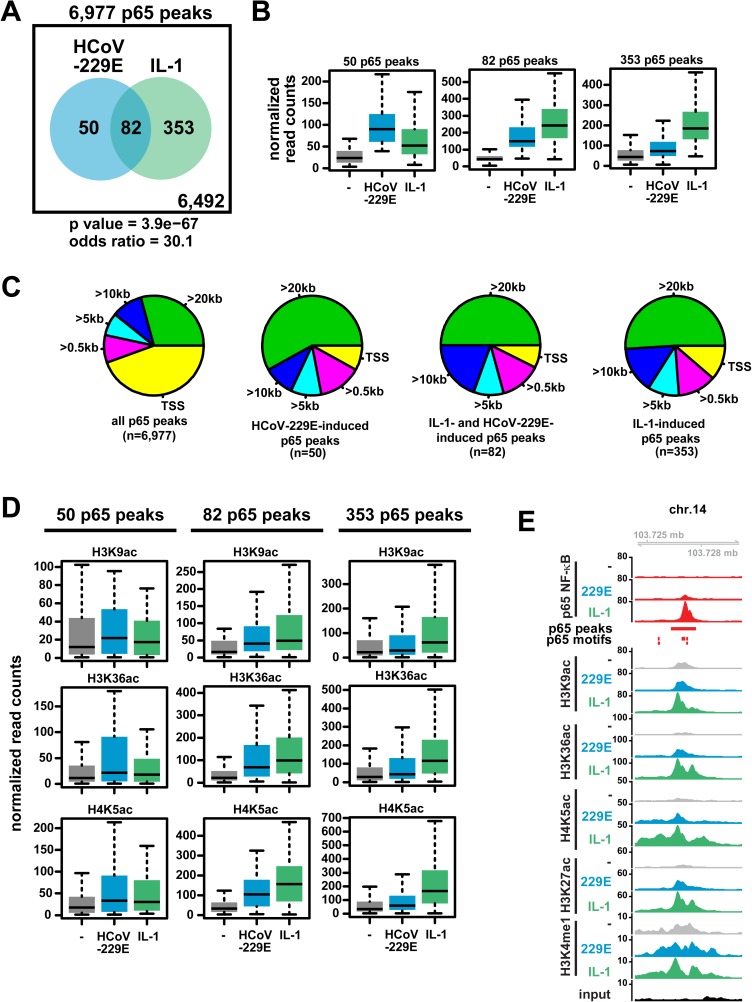
Genome-wide distribution and regulation of p65 NF-κB DNA binding sites in response to HCoV-229E compared to IL-1. (A) Total number of constitutive and HCoV-229E- or IL-1 regulated p65 ChIP-seq peaks. Regulation was defined by a ratio of normalized read counts derived from treated / untreated cells of at least 2-fold and a p value below 0.01. The likelihood of overlaps between HCoV-229E-or IL-1-regulated peaks occurring by chance is shown by the odds ratio and by the corresponding hypergeometric p value. (B) Quantification of normalized read counts for 50 HCoV-229E-specific, 353 IL-1-specific or 82 jointly regulated groups of p65 DNA-binding events. (C) Genome-wide localization pattern of p65 ChIP-seq peaks relative to the next annotated TSS. (D) Quantification of histone acetylation patterns of genomic regions containing regulated p65 peaks. (E) Examples of an intergenic p65 peak regulated by both HCoV-229E and IL-1, along with profiles of histone modifications observed in these cases. Shown are browser profiles of chr.14. The red horizontal bar marks the p65 peak region, vertical bars indicate predicted p65 DNA-binding motifs. Statistics for box plots (B, D) are shown in S4 Table. See also S7 and S8 Figs.

The ChIP-seq measurements also allowed us to address the genome-wide distributions of HCoV-229E- versus IL-1-regulated chromatin changes. In all instances, we detected significant overlaps between P(S5)-pol II recruitment and histone modifications after virus infection or cytokine treatment (S7 Fig). With respect to p65 NF-κB, 6,977 peaks were detected, of which 353 were regulated by IL-1, 82 were regulated by both IL-1 and HCoV-229E, and 50 by HCoV-229E only (Fig 7A). Quantification of p65 binding revealed much stronger signals for genome regions that were regulated specifically by IL-1 alone or regulated consistently by both IL-1 and HCoV-229E, whereas p65 peaks induced by HCoV-229E but not IL-1 had comparably lower signal intensities (Fig 7B). The majority of constitutive p65 peaks were localized close to promoter-TSS regions, whereas most of the regulated p65 peaks were located at more distant positions (> 20 kb away from the next TSS), suggesting that p65 is involved in occupying distant enhancer structures in these cases (Fig 7C). This conclusion is also supported by an increased acetylation of histones H3 and H4 in genome regions with increased p65 binding (Fig 7D). An example of such an IL-1- and HCoV-229E-regulated intergenic region, supported by inducible p65 recruitment to p65 DNA motifs combined with histone modifications in the same genomic region, is provided in Fig 7E. Taken together, the data lead us to conclude that, compared to the potent NF-κB activator IL-1, HCoV-229E causes a weak activation of the p65 NF-κB pathway at the chromatin level and regulates recruitment of this transcription factor to a small but specific group of mainly non-coding genomic regions.

### Regulation of genomic enhancers by HCoV-229E

**Fig 8:**
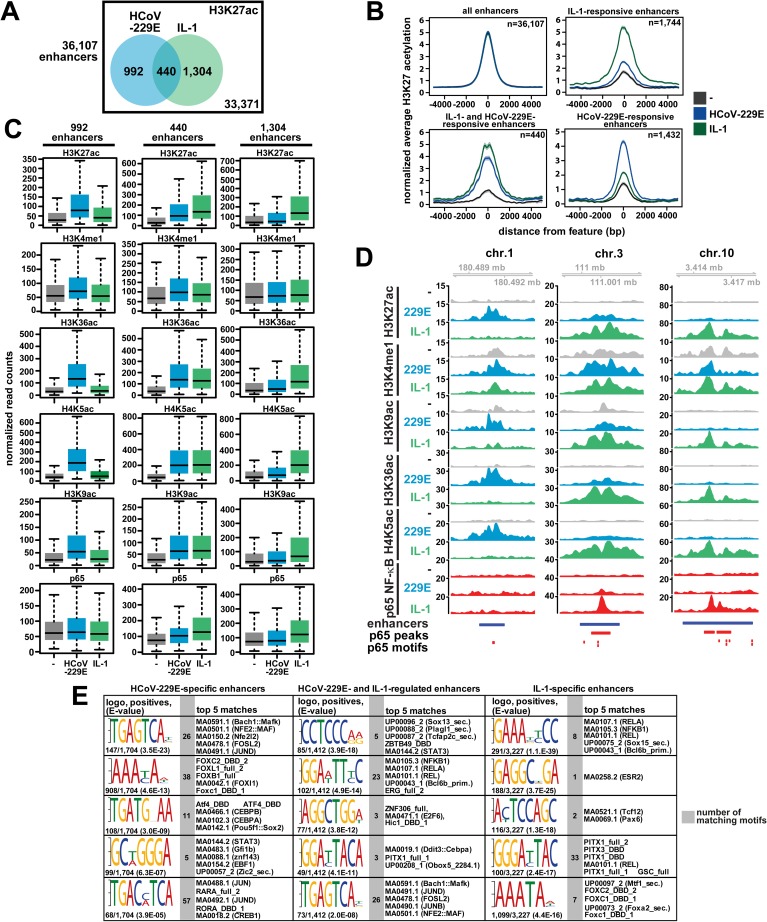
Identification and genome wide distribution of HCoV-229E-regulated enhancers. (A) Total number of active enhancers based on the presence of H3K27ac and H3K4me1. The Venn diagrams show numbers of regulated enhancers based on 2-fold change in H3K27ac in response to HCoV-229E, IL-1, or both. (B) Centered meta-profiles of H3K27ac across genomic enhancer regions as defined in (A). (C) Quantification of histone modifications and p65 binding for 992 HCoV-229E-specific and 1,304 IL-1-specific groups of enhancer regions as well as 440 groups of enhancer regions regulated by both stimuli. (D) Shown are browser profiles for enhancers representing HCoV-229E-specific (chr.1), IL-1-specific (chr.10) and jointly regulated (chr.3) enhancers. The blue bars mark the enhancer regions. Red bars mark p65 binding events and red vertical bars mark predicted p65-binding motifs. (E) Enhancer valleys defined as the intervening DNA regions between two H3K27ac peaks of all regulated enhancers as identified in Fig 8A were extracted revealing 1,704 regions specific for HCoV-229E, 1,412 regions regulated by both HCoV-229E and IL-1 and 3,227 regions regulated specifically by IL-1. These enhancer elements were then analyzed by DREME for the enrichment of DNA motifs followed by the analysis of matches to known TF binding sites by TomTom. Shown are the logos for the top five DREME-enriched motifs, the number of motifs per total number of identified enhancer valleys (positives) and the enrichment p value (E value) of the DREME analysis. The gray columns show the total numbers of matching motifs and the right columns show the top five TF-binding site motifs found by TomTom. See also S7 Fig. Statistics for box plots of (C) are shown in S4 Table. See also S7–S9 Figs.

**Fig 9:**
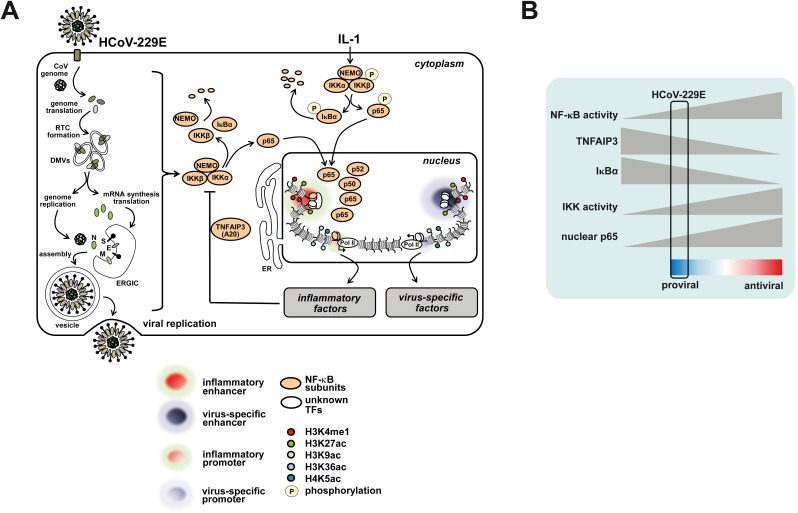
Schematic summaries of HCoV-229E-mediated chromatin and gene responses and attenuation of NF-κB activity. (A) Infection of cells with HCoV-229E causes partial degradation of IKKβ, NEMO and IκBα which results in limited nuclear translocation and chromatin recruitment of p65 and other NF-κB subunits and restricted activation of immune modulatory or inflammatory genomic enhancers and NF-κB target genes. The virus also activates a large repertoire of NF-κB-independent virus-specific enhancers and genes which are likely to regulate multiple signaling events and a diverse range of metabolic processes. (B) Results from this study further suggest that HCoV-229E fine tunes basal NF-κB activity at multiple levels to allow optimal replication and to prevent excessive expression of antiviral NF-κB target genes. For details see text. Abbreviations: DMV, double membrane vesicle; E, envelope protein; ERGIC, ER Golgi intermediate compartment; ER, endoplasmic reticulum; M, membrane protein; IκB, inhibitor of NF-κB; IKK, IκB kinase; NEMO, NF-κB essential modifier; N, nucleocapsid protein, RTC, replication-transcription complex; S, spike protein; TNFAIP3, Tumor necrosis factor alpha-induced protein 3.

These results raised the question of whether HCoV-229E infection leads to a more general activation, possibly affecting the enhancer repertoire across the entire HuH7 genome. To address this possibility, we determined the total number of active enhancers as assessed by the co-ocurrence of H3K4me1 and H3K27ac [28]. We found 36,107 regions carrying both histone marks (Fig 8A, S8A Fig). In total, 2,736 (7.6%) of all enhancers were identified to be regulated by IL-1 and/or HCoV-229E using a 2-fold change in H3K27ac as a threshold level (Fig 8A, sum of Venn diagrams). 1,432 and 1,744 enhancers, respectively, were specifically regulated by either HCoV-229E or IL-1, whereas 440 enhancers (16%) were found to be activated by both stimuli (Fig 8A). Meta-profiling showed that all regulated enhancers had low levels of H3K27ac prior to the activation and responded strongly with a symmetrical broadening of the H3K27ac peak following activation by IL-1 or HCoV-229E (Fig 8B). Compared to IL-1-specific enhancers, the HCoV-229E-specific enhancers were found to cover smaller regions of the genomic DNA, suggesting that these regions are occupied by a smaller number of DNA-binding factors and cofactors (Fig 8B). Quantitative assessment of histone modifications corroborated the specific increases of H3K27ac for all three groups of enhancers and little regulation of H3K4me1 (Fig 8C). It should be noted that all enhancer regions also showed additional regulation of H3K36ac, H4K5ac and H3K9ac. As these lysines are modified by different HATs such as CBP/p300 or GCN5 [29–31], this observation suggests that different HAT enzymes are recruited to and modify these structures (Fig 8C). Fig 8D provides 3 examples for each of the different enhancer groups. Notably, there was no virus-regulated p65 binding to the 992 HCoV-229E-specific or any of the IL-1-specific enhancers, suggesting that the vast majority of HCoV-229E enhancers is controlled by transcription factor combinations that differ from those employed by IL-1-regulated enhancers which always showed inducible p65 recruitment (Fig 8C and 8D). In line with this hypothesis, *de novo* motif searches revealed a complex assembly of *cis*-elements that were characteristic for each of the 3 regulated groups of enhancers (Fig 8E). These structures matched to a large number of known transcription factor-binding sites (Fig 8E). While the HCoV-229E-specific enhancers are predicted to bind various combinations of AP-1 subunits (Jun, JunD, FOSL2, ATF4) and C/EBPs, the IL-1 regulated enhancers are enriched for NF-κB and alternative combinations of AP-1 sites (Fig 8E). Additionally, a variety of other TFs, such as FOXC2, FOXL1, FOXB1, DDIT3 and others, can bind to these sites in different combinations (Fig 8E). Thus, the three groups of enhancers provide composite binding sites for stimulus-specific combinations of TFs. Accordingly, GO classifications of all annotated genes next to the HCoV-229E-specific enhancers revealed an enrichment for catabolic and ER stress pathways, whereas genes next to enhancers regulated by both IL-1 and HCoV-229E or by IL-1 alone are highly enriched for multiple immunoregulatory pathways (S8B Fig). ChIP-PCR experiments using PHA-408 confirmed that the two enhancers on Chr.1 and on Chr.10 shown in Fig 8D differ in their virus- or IL-1-mediated p65 recruitment and sensitivity to IKKβ inhibition (S9 Fig). In conclusion, these data reveal that HCoV-229E infection activates specific histone modification patterns in a large number of genomic regions that are likely to orchestrate the genome wide gene response to this virus. Apart from a number of shared enhancer regions, this pattern is fundamentally different from the prototypical NF-κB p65-driven IL-1 enhancer structures and provides an explanation for the large divergent sets of virus-specific genes (summarized in Fig 9A).

## Discussion

Emerging or re-emerging RNA viruses cause significant morbidity and mortality in humans and animals [1, 32, 33]. With few exceptions, these RNA viruses complete their viral life cycle in the cytoplasm but need to reprogram and/or adjust specific biosynthetic and other pathways required for viral replication and production of infectious virus progeny according to their specific requirements [34, 35]. Simultaneously, intrinsic host cell defense systems are activated and, in many cases, counteracted by a multitude of strategies that viruses developed during viral evolution [32]. Despite significant scientific progress in the past few years, the complete spectrum of interactions between plus-strand RNA viruses and their hosts is far from being understood, in particular if it comes to understanding complex regulatory networks at a genome-wide level [8, 36]. Here, we provide a high-resolution view of both transcriptome and chromatin changes in cells infected with the alphacoronavirus HCoV-229E, a virus that causes mild upper respiratory tract disease and is efficiently transmitted in the human population, indicating excellent adaption to its human host. The sophisticated fine-tuning of IKK-NF-κB activity and the identification of thousands of coronavirus-specific enhancers reported in this study provides important insight into the enormous complexity by which an RNA virus resets the host cell chromatin response to change expression levels of many hundreds of host cell genes and, at the same time, stimulates future studies into the molecular interplay between corona- and other RNA viruses and their hosts.

There is conflicting evidence regarding the activation or importance of the NF-κB pathway for human CoV [4, 37–40]. In our view, this may be attributed to the methods used to study NF-κB activation or the ectopic expression of viral proteins rather than infection with intact virus but may also be linked to biological differences between CoV that belong to different virus species/genera and are known to infect different hosts. Here, we provide the first in-depth and high-resolution analysis of endogenous NF-κB activation and p65 DNA binding in virus-infected cells. In addition, we compared the HCoV-229E-triggered responses directly to those elicited by IL-1, a potent inducer of the classical NF-κB pathway. Our data suggest two different consequences of HCoV-229E infection for NF-κB activity: On the one hand the viral infection leads to NF-κB activation, as seen by IκBα degradation, p65 chromatin recruitment and the inducible transcription of NF-κB target genes. This elevated NF-κB activity has a pro-viral function, as evidenced by impaired virus replication after IKK inhibition or p65 knockdown. Mechanistically, elevated NF-κB activity also allows for synthesis of the A20 protein which is required for efficient virus replication. These findings are in line with the recent observation that deletion of the A20-encoding gene protects mice from influenza A virus infection [41, 42]. On the other hand, we also observed that HCoV-229E has acquired specific mechanisms to dampen NF-κB activity, as evidenced by lower levels of p65 chromatin recruitment and comparably low induction of gene expression. HCoV-229E infection failed to induce IKK phosphorylation and only caused insufficient phosphorylation and incomplete degradation of IκBα. In addition, the steady state levels of NEMO and IKKβ decreased during infection which could be due to several mechanisms including phosphorylation of eIF2α to shut off protein *de novo* synthesis [43, 44]. The simultaneous induction and restriction of the NF-κB response is typically seen for many different RNA viruses [20]. This mechanism is visualized in Fig 9B and presumably serves to ensure a corridor of elevated NF-κB activity allowing to support viral replication, while also preventing full activity of the transcription factor that counteracts virus infection by enabling the synthesis of anti-viral mediators such as IFN or other cytokines. This could also explain the pro-viral function of A20, as it prevents exaggerated anti-viral gene expression and NF-κB activity.

At the level of gene expression we found that the HCoV-229E-regulated gene pattern is overlapping with the genes affected by the zoonotic virus SARS-CoV including IL-6 and several chemokines [4, 6]. To our knowledge, none of these earlier studies addressed the question of whether the observed changes of host cell gene expression were caused by the replicating virus itself or (partly or largely) by factors potentially present in the virus preparation used to infect/inoculate the cells to be used for subsequent analyses. Using appropriate controls and two different human cell lines, we were able to identify a common set of genes that is specifically induced in productively infected cells with ongoing HCoV-229E genome replication and expression. This gene set contains factors involved in the ER stress response, consistent with an earlier study showing that some genes from this group (*PERK*, *ATF3/4*, *ERO1α*) are induced at the protein level in IBV-infected avian cells [23, 45, 46]. Our mRNA expression and bioinformatics analysis identified a large number of additional genes of the ER stress response that are induced by HCoV-229E, such as CHAC1, an enzyme that degrades glutathione [47, 48] and others (*FICD*, *HERPUD1*, *DNAJB9/ERDJ4*) functioning as regulators of AMPylation, ubiquitylation or co-chaperones, respectively [49–51]. Moreover, HCoV-229E was found to upregulate transcription factors (TFs) with known functions such as EGR1 and c-JUN, but also poorly characterized TFs such as zinc finger (ZNF)165, basic helix-loop-helix-type transcription factors (BHLHE40/41) or zinc finger and BTB domain-containing 43 (ZBTB43) [52, 53].

In an attempt to dissect virus-specific effects from well-characterized inflammatory stimuli, we performed a detailed comparison of HCoV-229E-and IL-1-regulated genes and were able to identify a common set of genes that appears to be regulated by both HCoV-229E and IL-1. This small group of genes mainly involves factors involved in innate immune regulation or cytokine functions (e.g. *CXCL2*, *CXCL1*, *CXCL5*, *IL8*, *GDF15)* or MAPK pathway signaling (e.g. *DUSP1*, *8*, *10*, *16*, *c-JUN*, *JUN D*), and helps explain the inflammatory phenotype that, in many cases, is associated with coronavirus infections. Altogether, our comprehensive analysis of the HCoV-229E-regulated transcriptome provides numerous candidate genes that warrant further functional investigations to better understand their potential role in the viral life cycle, host defense mechanisms or other pathways.

A detailed analysis of the recruitment pattern of RNA pol II and phosphorylated pol II revealed that about 200 HCoV-229E-induced genes are strongly upregulated at the transcriptional level. 49 of these genes were shared with IL-1, a stimulus that very potently activates P(S5)-pol II binding to its target genes [24]. Furthermore, the data support the idea that, at these loci, HCoV-229E infection reorganizes the chromatin structure at or near promoter regions, TSS and gene bodies, for example by increasing acetylation of H3K36 and H4K5, suggesting that virus-induced host cell responses strictly depend on nuclear transcriptional mechanisms.

The restricted chromatin targeting of p65 by HCoV-229E may also involve specific cooperative interactions with other eukaryotic TFs, such as MAPK- or ER stress-activated AP-1 proteins, interactions with upregulated corepressors such as ANKRD1, or interactions with the nuclear pool of N proteins. In line with this idea, ANKRD1 was recently described to suppress NF-κB activity, whereas viral N proteins have consistently been reported to translocate to the nucleus, although to date there is no evidence for an involvement of the N protein in chromatin processes [26, 54, 55].

Additional support for HCoV-229E-specific TF complexes comes from experiments conducted to characterize the enhancer repertoire of virus-infected cells. A striking finding of our study is the identification of more than 1,000 enhancers that are activated specifically by HCoV-229E. The lack of p65 recruitment and the discovery of specifically enriched motifs for specific groups of TFs distinguish these structures from the IL-1-regulated enhancers. These motifs contain binding sites for a number of AP-1 proteins (FOSL2, JUND, c-JUN) that are downstream of the ERK or JNK pathways known to be triggered by SARS-CoV or IBV infection [23, 56]. Another abundantly found motif is bound by various members of the forkhead superfamily of TFs (FOXC1/2, FOXL1, FOXB1). These multifunctional proteins regulate transcriptional programs in cancer and innate immunity but their roles in RNA virus infection are unknown and await further investigation [57, 58]. Additionally, the motif TGATGXAA is found in 108 virus-specific enhancers (Fig 8E). This sequence matches the consensus sequence for the C/EBP-ATP response element CARE (TGATGXAAX) [59]. The CARE element is crucial for the ATF4-dependent activation of a specific set of genes that are (up)regulated during amino acid starvation [60]. ATF4 and its heterodimerization partners of the C/EBP or JUN families of TFs control metabolic gene expression programs but also the initiation of cell death upon activation of PERK-dependent phosphorylation of eIF2α during the ER stress response [61–63]. Thus, the large enhancer repertoire identified in this study likely coordinates a global gene response to cope with increased translational demand and the elevated load of misfolded proteins in the ER during viral replication. Altogether, the identification in non-coding genomic regions of three types of enhancers that respond to virus, to IL-1, or to both against a background of 90% of all the other enhancer regions that did not change in response to these stimuli, provides an impressive example of the versatile usage of a small portion of the enhancer repertoire of a human cell to trigger stimulus-specific gene expression [14–16].

In conclusion, our results provide comprehensive insight into host cell transcriptome changes induced by HCoV-229E infection and link this information to the underlying chromatin changes as summarized in Fig 9. We also show novel mechanisms ensuring the induction of a well-balanced and self-limiting NF-κB response to support viral propagation.

## Materials and methods

### Cell culture and virus preparations

A549 human alveolar basal epithelial cells and HeLa cells (both ATCC) and HuH7 human hepatoma cells (Japanese Collection of Research Bioresources (JCRB) cell bank) [64] were maintained in Dulbecco’s modified Eagle’s medium (DMEM), complemented with 10% fetal calf serum (FCS), 2 mM L-glutamine, 100 U/ml penicillin and 100 μg/ml streptomycin. The genome sequence of the human coronavirus (strain 229E) used in this study is available from GenBank (accession number 304460). Infections of A549, HeLa and HuH7 cells were performed at 33°C using the indicated multiplicities of infection (MOI). Virus titers (TCID_50_/ml) were determined using HuH7 cells. IL-1 treatment was done at 33°C using identical conditions and cell culture medium.

### RT-qPCR, microarray analyses and bioinformatics

Quantitative analysis of mRNA expression of individual A549 or HuH7 or viral genes was performed as described [24]. Transcriptomes were determined using Agilent 60k microarrays followed by KEGG pathway analysis of regulated gene sets determined by overrepresentation or gene set enrichment analyses.

### Laser microdissection and fluorescence microscopy

Cells expressing viral N protein were excised with a Leica LMD6000 system. Then, total RNA was extracted and subjected to RT-qPCR or microarray analysis. Indirect immunofluorescence analyses were performed on Leica DMIRE2 or DMi8 fluorescence microscopes as described [65].

### Preparation of cell extracts and immunoblotting

Expression, phosphorylation and subcellular distribution of cellular proteins were analyzed as described [24].

### ChIP-PCR, Chip-seq and bioinformatics

Protein:DNA complexes were cross-linked *in vivo* by formaldehyde treatment, immunoprecipitated from denatured cell extracts and enriched DNA fragments were purified and quantified by ChIP-PCR or deep DNA sequencing. ChIP-seq reads were mapped to the human genome (built HG19), binding events were normalized to input samples and differential binding of phosphorylated RNA polymerase or p65 or changes in histone acetylation events were quantified and further analyzed by bioinformatics as described [24].

### Statistics

Statistics (Mann-Whitney Rank, Wilcoxon signed rank or t-tests) were calculated using R, SigmaPlot11, GraphPadPrism6.0 and MS EXCEL2010.

Complete experimental procedures including reagents, buffers, nucleotide sequences, additional methods and software used are described in detail in S1 Supporting Experimental Procedures.

Microarray (GSE89167) and ChIP-seq (GSE89212) data have been deposited at geo@ncbi.nlm.nih.gov.

## Supporting information

S1 Fig Related to Fig 1. HCoV-229E virus replication in A549 cells. (A) Expression of HCoV-229E genomic RNAs (RNA1 encoding *nsp8* and RNA2 encoding s*pike*) in cells infected with replicating HCoV-229E or inactivated versions of HCoV-229E for different length of time. Shown are the means +/- s.e.m. of a technical replicate from a representative experiment. (B) An example of the immunofluorescence and laser microdissection procedure that was used to isolate the cell populations described in the experiments shown in Fig 1B–1E. The scale bar is 100 μm.
(TIF)

S2 Fig Related to Fig 1. KEGG pathway analysis of genes differentially expressed in HCoV-229E-infected A549 cells. (A) Overrepresentation analysis (ORA, blue colors) of differentially expressed genes (upper panels) and gene set enrichment analyses (GSEA, green, red and yellow colors) using the entire set of expressed genes (lower panels) were used to identify pathways deregulated in HCoV-229E-infected cells as assessed by the microarray experiments shown in Fig 1. Bubble plots show significance of enriched pathway components at the Y-axis, the percentage of genes of each pathway that were found to be expressed at the X-axis, the number of pathway components symbolized by size of the bubbles and the direction of gene regulation by red, yellow and green colors. (B) Expressed genes from the LMD microarray experiments shown in Fig 1B–1D were mapped to the most often deregulated KEGG pathways 04141 (protein processing in endoplasmic reticulum) and 03013 (RNA transport) as shown in (A). Colors show direction of gene regulation.
(TIF)

S3 Fig Related to Fig 2. HCoV-229E-induced genes in HuH7 cells. (A) Time course experiments were performed and the mRNA expression of the indicated genes was analyzed by RT-qPCR. Data show means +/- s.e.m. from three (uninfected, HCoV-229E) or two (HCoV-229E_inactive_) independent experiments. (B) Depicted are all individual ratio values for the right heatmap shown in Fig 2B representing genes induced by HCoV-229E only but not by IL-1 (log_2_ ratios ≤ 0.7). Ratio values were calculated by dividing the expression values of each individual microarray experiment by the mean signals of the four control experiments. Additionally, results from microarray experiments of infections using heat-inactivated HCoV-229E (inactive) are displayed.
(TIF)

S4 Fig Related to Figs 2 and 3. Gene expression, RNA pol II recruitment and histone modifications in HuH7 cells infected with HCoV-229E or treated with IL-1. (A) HuH7 cells were infected for 24 h or treated with IL-1 for 1 h and the mRNA expression of the indicated genes was determined by RT-qPCR. Data show means +/- s.e.m. from two (*CHAC1*, *HERPUD1*), three (*TNFAIP3*), or at least eight independent experiments. (B) The recruitment of RNA polymerase II and P(S5)-pol II was determined by ChIP-PCR. IgG ChIP experiments served as controls. Data show means +/- s.e.m. from three independent experiments. (C) Quantification of histone modifications and P(S5)-pol II recruitment for the genes shown in Fig 2D. Normalized cumulative read counts were compiled 2 kB up- and downstream of the TSS as shown in Fig 3A.
(TIF)

S5 Fig Related to Figs 4 and 5. Regulation of IKK complex subunits and of IκBα at the mRNA level. (A) HuH7 cells were infected for 24 h with HCoV-229E or heat-inactivated virus or were treated with IL-1 for 1 h. Then, the levels of mRNAs encoding IKKα (*IKBKA/CHUK*), IKKβ (*IKBKB*), NEMO (*IKBKG*) or IκBα (*NFKBIA*) were determined. Data show means +/- s.e.m. from at least four independent experiments. (B, C) ChIP-seq tracks of the *NFKBIA* locus are shown. Details of these experiments are described in the legends of Figs 3 and 5.
(TIF)

S6 Fig Related to Fig 5. Subcellular distributions of host cell proteins in HCoV-229E-infected or IL-1-treated HuH7 or HeLa cells. (A) Immunoblots from experiments using HuH7 cells were performed as described in the legend of Fig 5A. Membranes were probed with an additional antibody recognizing p65 NF-κB or with antibodies against the c-REL NF-κB subunit. Viral replication and purity of fractions were confirmed using antibodies against N protein, tubulin and RNA polymerase II. (B, C) HuH7 (B) or HeLa (C) cells were analyzed by indirect immunofluorescence for viral infection rate and subcellular distribution of p65 NF-κB using antibodies against N protein or p65, respectively. IF-control indicates omission of primary antibodies to judge the specificity of the fluorescence signals. Ph, phase contrast. The scale bars are 25 μm.
(TIF)

S7 Fig Related to Figs 7 and 8. Total peak numbers and overlaps of regulated genomic regions from ChIP-seq experiments assessing histone modifications and recruitment of P(S5)-pol II in HuH7 cells. Shown are the total numbers of peaks for histone modifications and P(S5)-polymerase II recruitment. Numbers for peaks regulated by HCoV-229E or IL-1 were derived based on differences of at least 2-fold and a p value below 0.05. The likelihood of overlapping regulated peaks occurring by chance is shown by odds ratios and by the corresponding hypergeometric p values.
(TIF)

S8 Fig Related to Figs 7 and 8. ChIP-seq profiles across a gene-rich non-regulated genomic region and GO annotation of enhancer-associated genes. (A) Shown is an example for all ChIP-seq data obtained for HuH7 cells in this study showing non-regulated enhancers (blue bars), regions of constitutive P(S5)-pol II recruitment (gray bars), NF-κB binding (red bars) and predicted NF-κB motifs (vertical red bars). (B) Gene Ontology (GO) analyses for all annotated genes located next to the three groups of enhancers described in Fig 8C. Differentially up-regulated enhancers (as detected by > 2-fold induction of H3K27ac binding) were analyzed for over-represented GO terms amongst the genes mapped to respective enhancer intervals. Bar graphs show negative decadic logarithms of the binomial p values of significantly enriched GO terms.
(TIF)

S9 Fig Related to Fig 8. The IKKβ inhibitor PHA-408 suppresses histone modifications and p65 recruitment at HCoV-229E- or IL-1-regulated enhancers. Chromatin prepared from HuH7 cells treated exactly as described in the legend of Fig 5E was used to determine by ChIP experiments the histone modifications, p65 recruitment and histone densities at the virus-specific enhancer region on Chr.1 or the IL-1-specific enhancer region on Chr. 10 shown in Fig 8D. Shown are the results from two independent ChIP-PCR experiments, IgG immunoprecipitations served as negative control.
(TIF)

S1 Supporting Experimental Procedures (PDF)

S1 Table Contains data belonging to Fig 1A. (XLSX)

S2 Table Contains data belonging to Fig 1C. (XLSX)

S3 Table Contains data belonging to Fig 2C. (XLSX)

S4 Table Contains statistics for Fig 7B and 7D and Fig 8C. (XLSX)
