# Supplementary figures and images for: The NF-κB-dependent and -independent transcriptome and chromatin landscapes of human coronavirus 229E-infected cells
Source: PLoS Pathog. 2017 Mar 29;13(3):e1006286. doi: 10.1371/journal.ppat.1006286 (PMC5386326; doi:10.1371/journal.ppat.1006286)

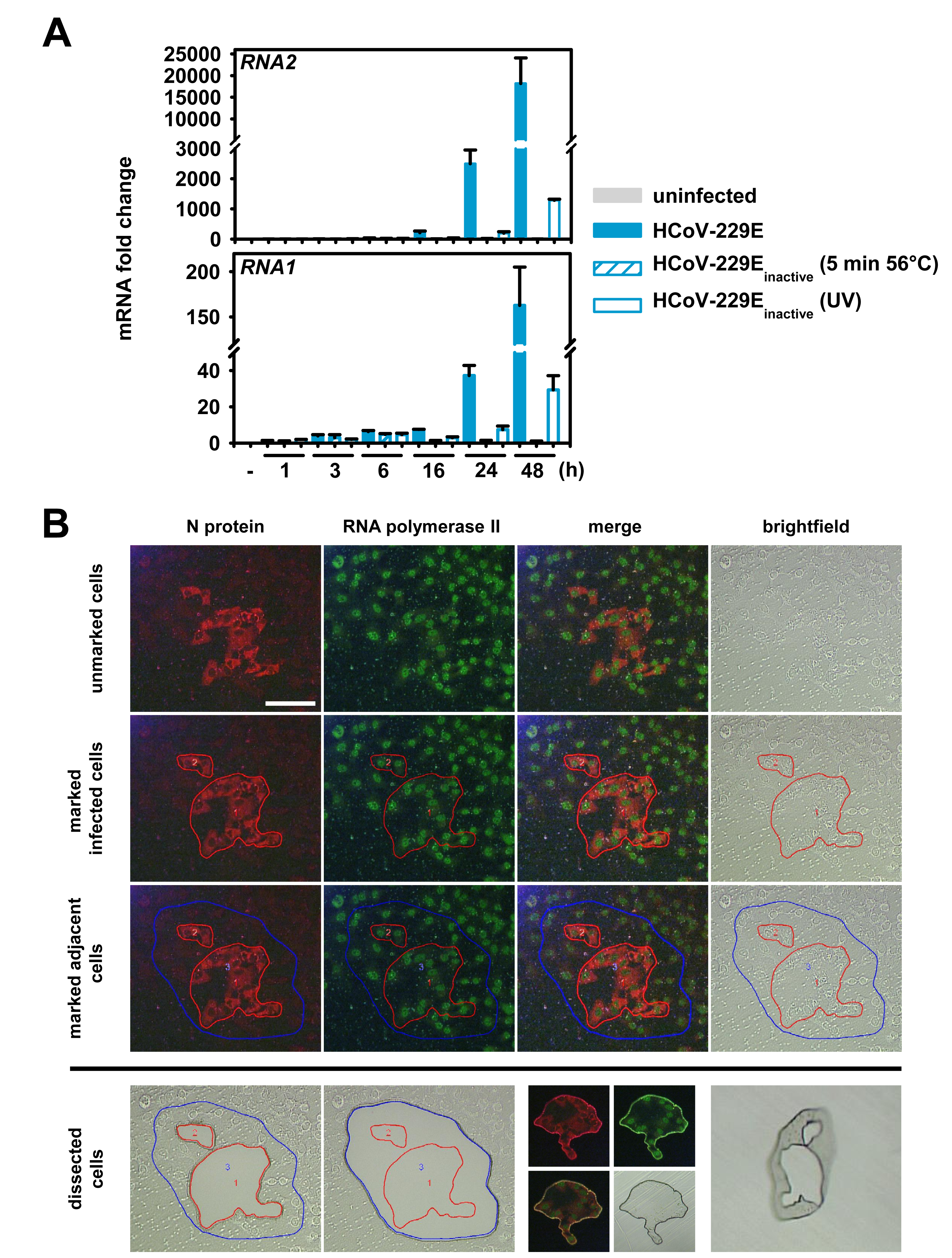

Supplement: S1 Fig — (A) Expression of HCoV-229E genomic RNAs (RNA1 encoding nsp8 and RNA2 encoding spike) in cells infected with replicating HCoV-229E or inactivated versions of HCoV-229E for different length of time. Shown are the means +/- s.e.m. of a technical replicate from a representative experiment. (B) An example of the immunofluorescence and laser microdissection procedure that was used to isolate the cell populations described in the experiments shown in Fig 1B–1E. The scale bar is 100 μm. (TIF) [file ppat.1006286.s001.tif]

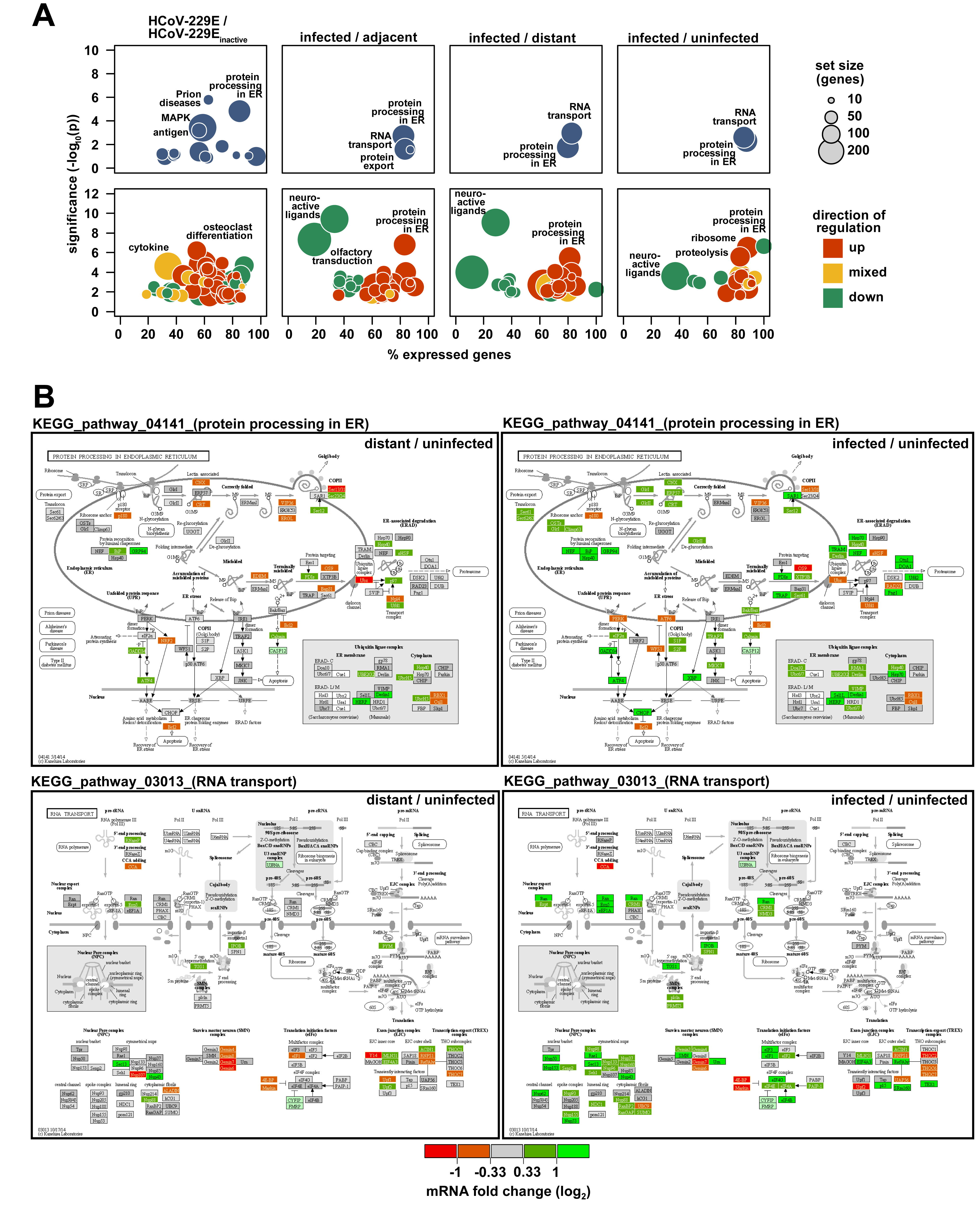

Supplement: S2 Fig — (A) Overrepresentation analysis (ORA, blue colors) of differentially expressed genes (upper panels) and gene set enrichment analyses (GSEA, green, red and yellow colors) using the entire set of expressed genes (lower panels) were used to identify pathways deregulated in HCoV-229E-infected cells as assessed by the microarray experiments shown in Fig 1. Bubble plots show significance of enriched pathway components at the Y-axis, the percentage of genes of each pathway that were found to be expressed at the X-axis, the number of pathway components symbolized by size of the bubbles and the direction of gene regulation by red, yellow and green colors. (B) Expressed genes from the LMD microarray experiments shown in Fig 1B–1D were mapped to the most often deregulated KEGG pathways 04141 (protein processing in endoplasmic reticulum) and 03013 (RNA transport) as shown in (A). Colors show direction of gene regulation. (TIF) [file ppat.1006286.s002.tif]

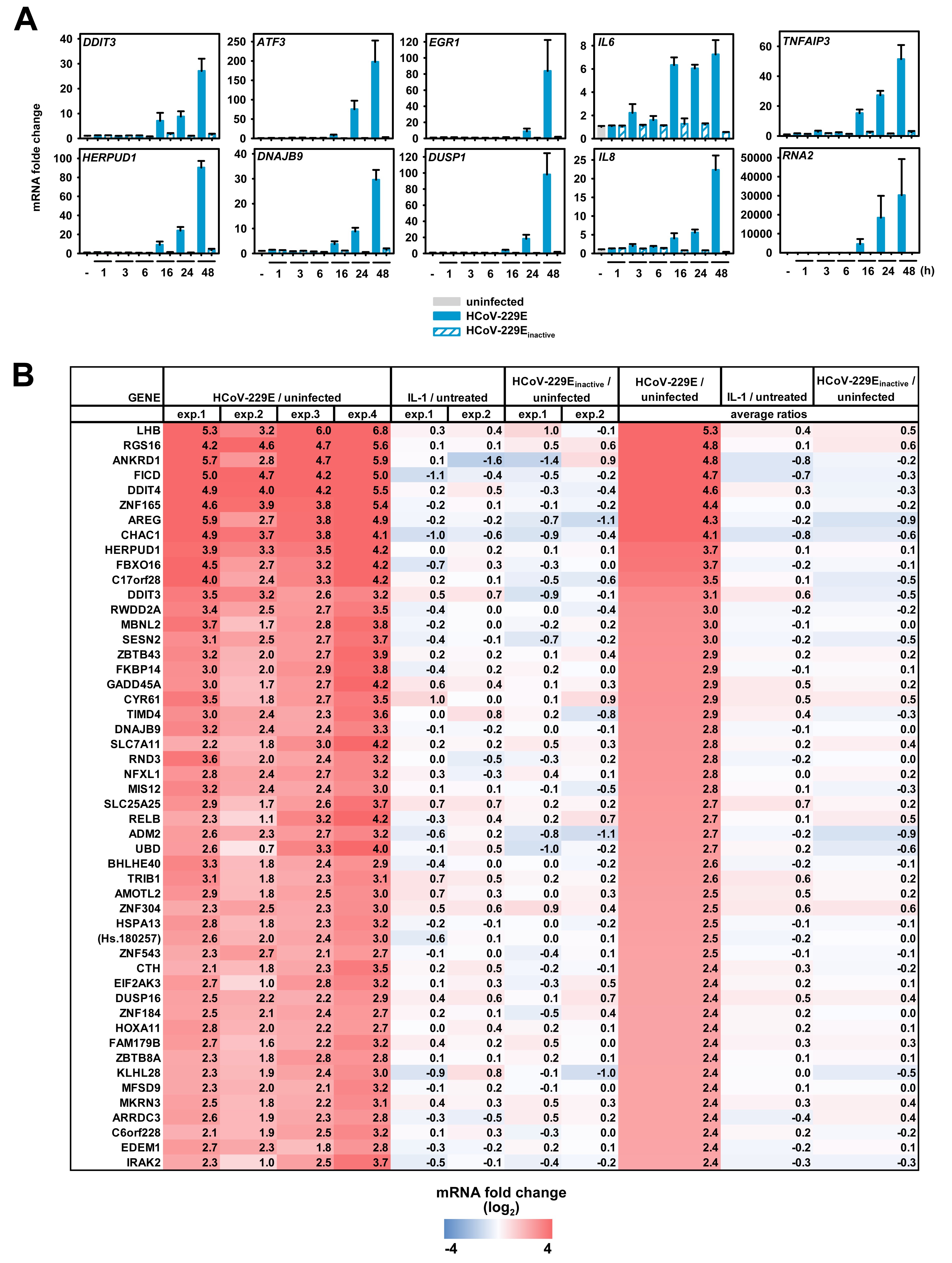

Supplement: S3 Fig — (A) Time course experiments were performed and the mRNA expression of the indicated genes was analyzed by RT-qPCR. Data show means +/- s.e.m. from three (uninfected, HCoV-229E) or two (HCoV-229Einactive) independent experiments. (B) Depicted are all individual ratio values for the right heatmap shown in Fig 2B representing genes induced by HCoV-229E only but not by IL-1 (log2 ratios ≤ 0.7). Ratio values were calculated by dividing the expression values of each individual microarray experiment by the mean signals of the four control experiments. Additionally, results from microarray experiments of infections using heat-inactivated HCoV-229E (inactive) are displayed. (TIF) [file ppat.1006286.s003.tif]

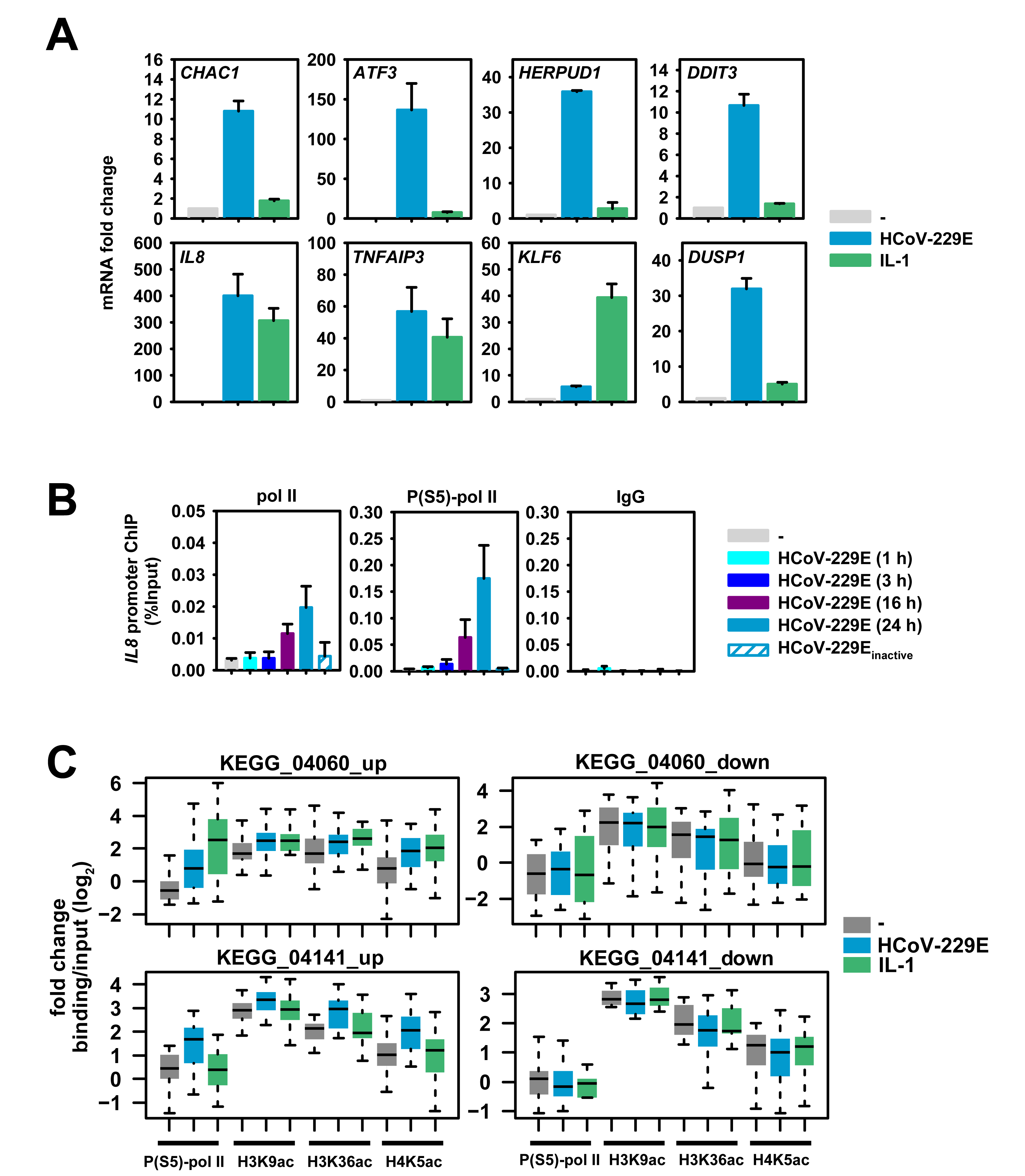

Supplement: S4 Fig — (A) HuH7 cells were infected for 24 h or treated with IL-1 for 1 h and the mRNA expression of the indicated genes was determined by RT-qPCR. Data show means +/- s.e.m. from two (CHAC1, HERPUD1), three (TNFAIP3), or at least eight independent experiments. (B) The recruitment of RNA polymerase II and P(S5)-pol II was determined by ChIP-PCR. IgG ChIP experiments served as controls. Data show means +/- s.e.m. from three independent experiments. (C) Quantification of histone modifications and P(S5)-pol II recruitment for the genes shown in Fig 2D. Normalized cumulative read counts were compiled 2 kB up- and downstream of the TSS as shown in Fig 3A. (TIF) [file ppat.1006286.s004.tif]

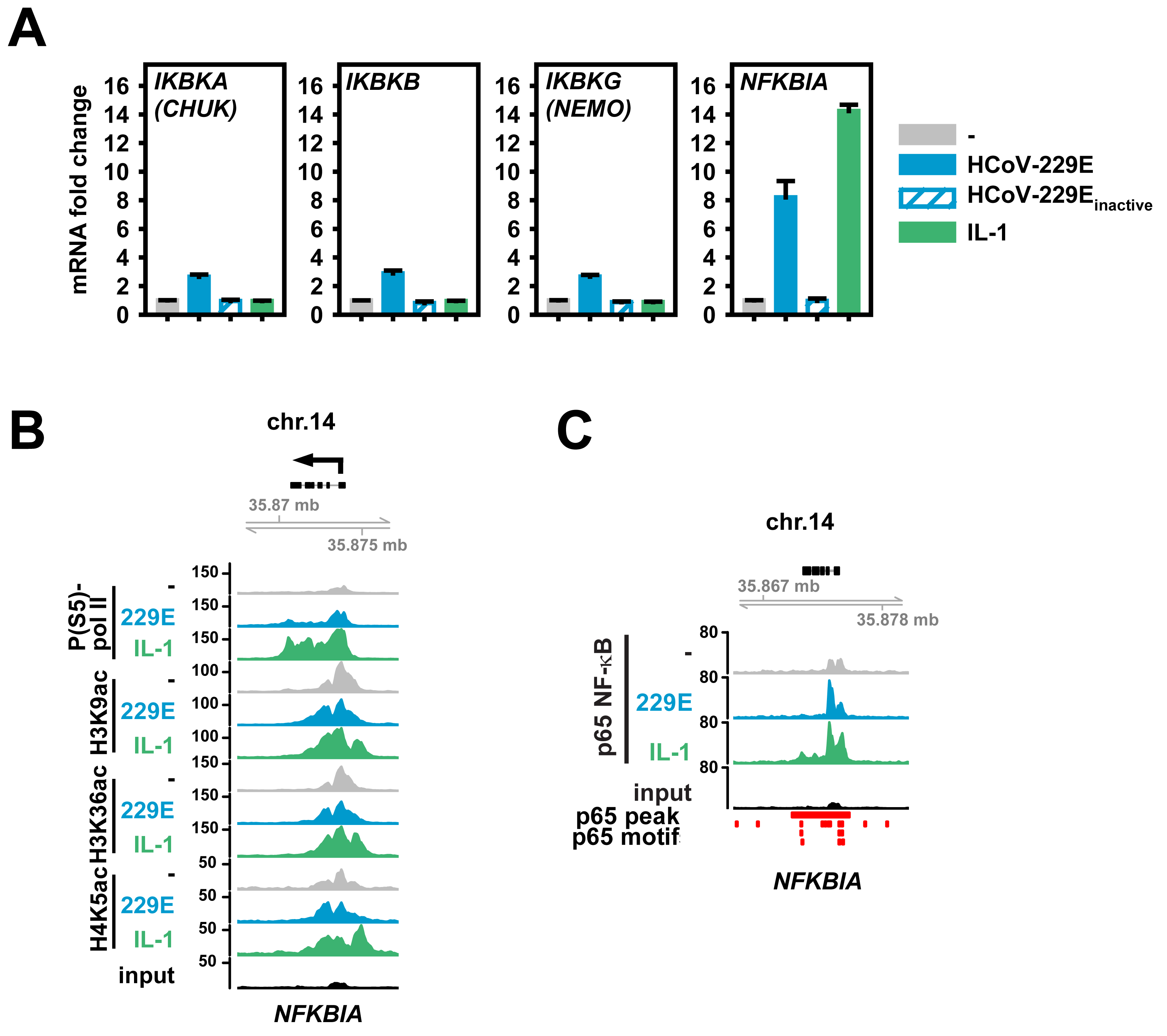

Supplement: S5 Fig — (A) HuH7 cells were infected for 24 h with HCoV-229E or heat-inactivated virus or were treated with IL-1 for 1 h. Then, the levels of mRNAs encoding IKKα (IKBKA/CHUK), IKKβ (IKBKB), NEMO (IKBKG) or IκBα (NFKBIA) were determined. Data show means +/- s.e.m. from at least four independent experiments. (B, C) ChIP-seq tracks of the NFKBIA locus are shown. Details of these experiments are described in the legends of Figs 3 and 5. (TIF) [file ppat.1006286.s005.tif]

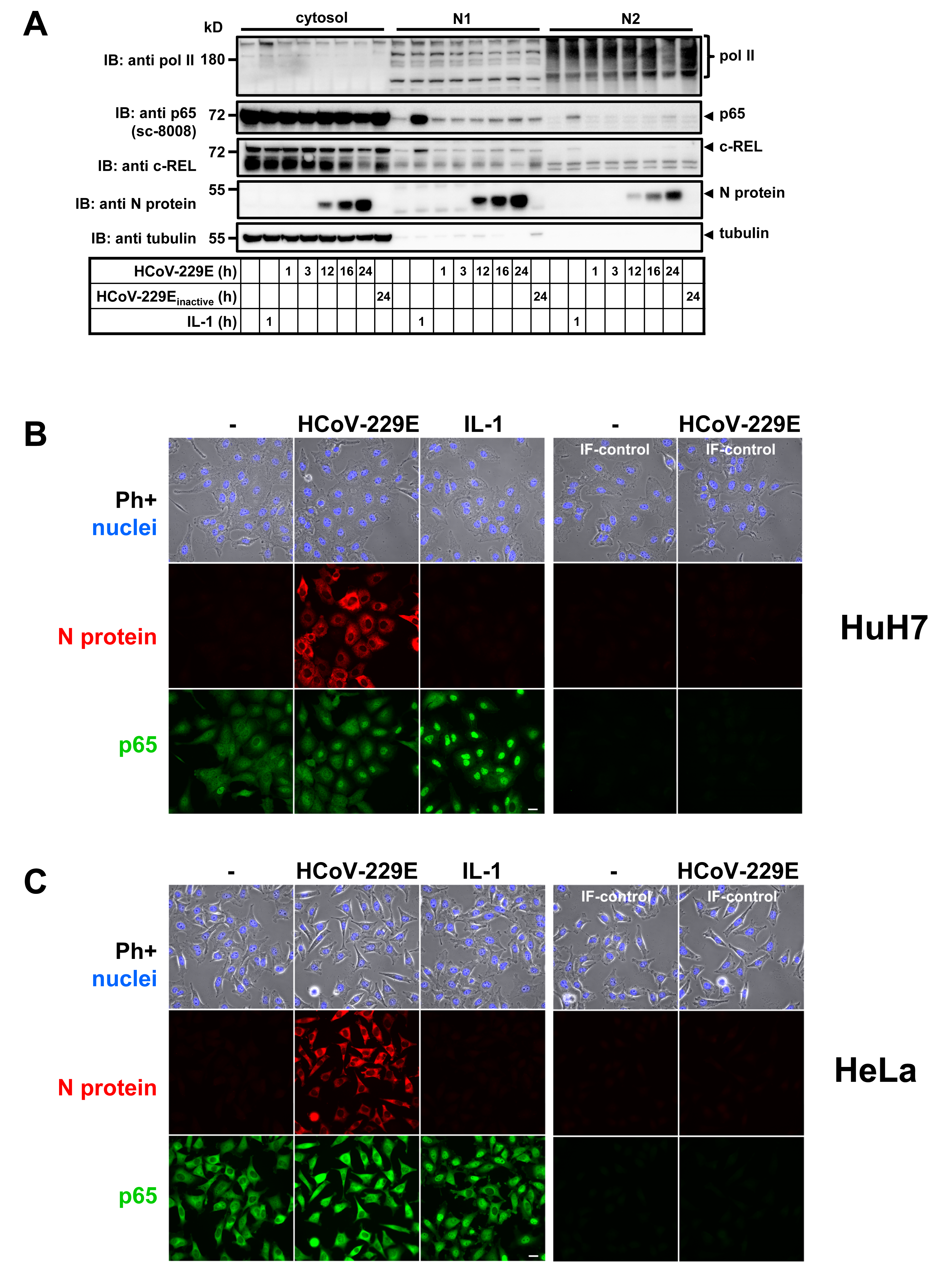

Supplement: S6 Fig — (A) Immunoblots from experiments using HuH7 cells were performed as described in the legend of Fig 5A. Membranes were probed with an additional antibody recognizing p65 NF-κB or with antibodies against the c-REL NF-κB subunit. Viral replication and purity of fractions were confirmed using antibodies against N protein, tubulin and RNA polymerase II. (B, C) HuH7 (B) or HeLa (C) cells were analyzed by indirect immunofluorescence for viral infection rate and subcellular distribution of p65 NF-κB using antibodies against N protein or p65, respectively. IF-control indicates omission of primary antibodies to judge the specificity of the fluorescence signals. Ph, phase contrast. The scale bars are 25 μm. (TIF) [file ppat.1006286.s006.tif]

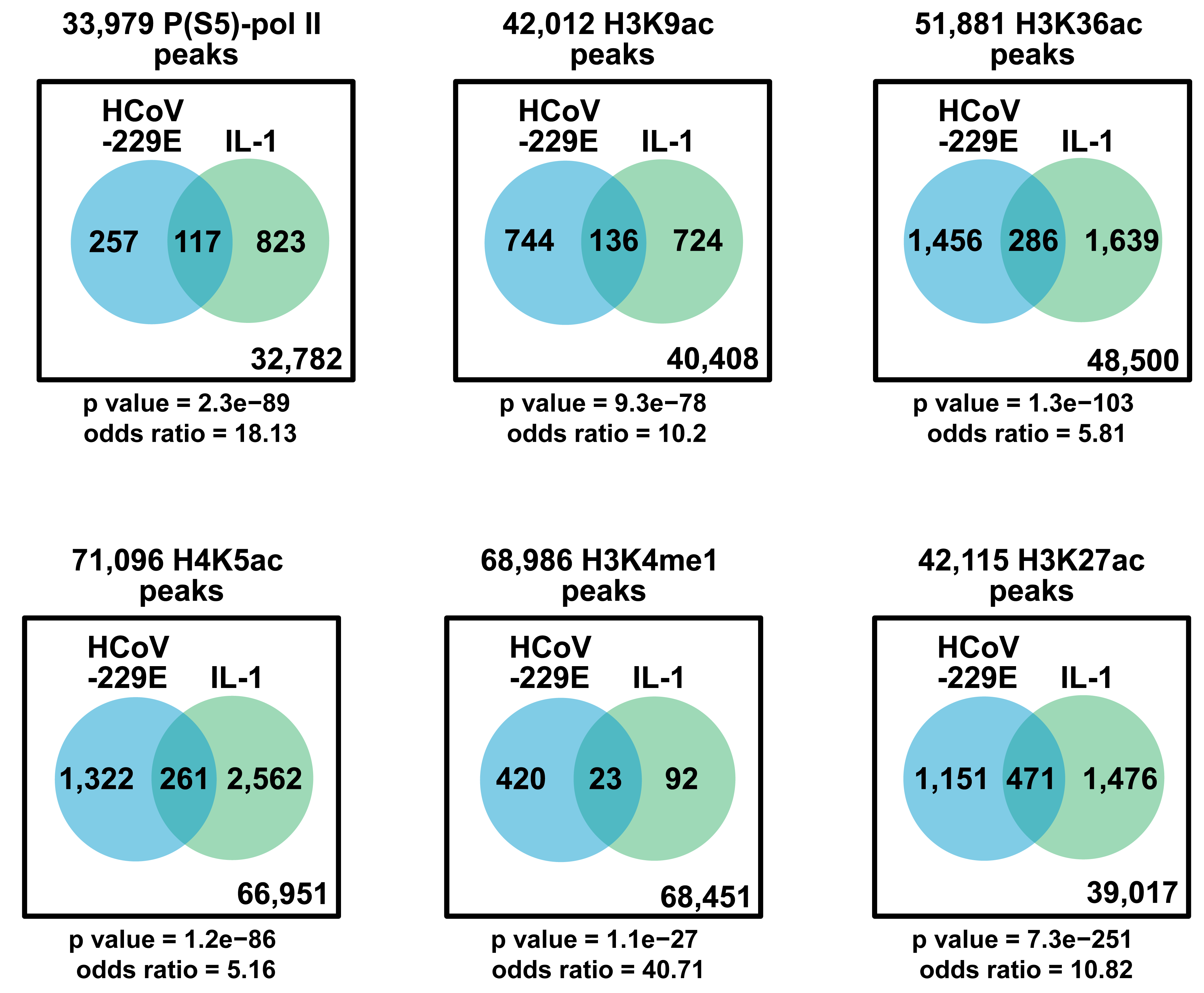

Supplement: S7 Fig — Shown are the total numbers of peaks for histone modifications and P(S5)-polymerase II recruitment. Numbers for peaks regulated by HCoV-229E or IL-1 were derived based on differences of at least 2-fold and a p value below 0.05. The likelihood of overlapping regulated peaks occurring by chance is shown by odds ratios and by the corresponding hypergeometric p values. (TIF) [file ppat.1006286.s007.tif]

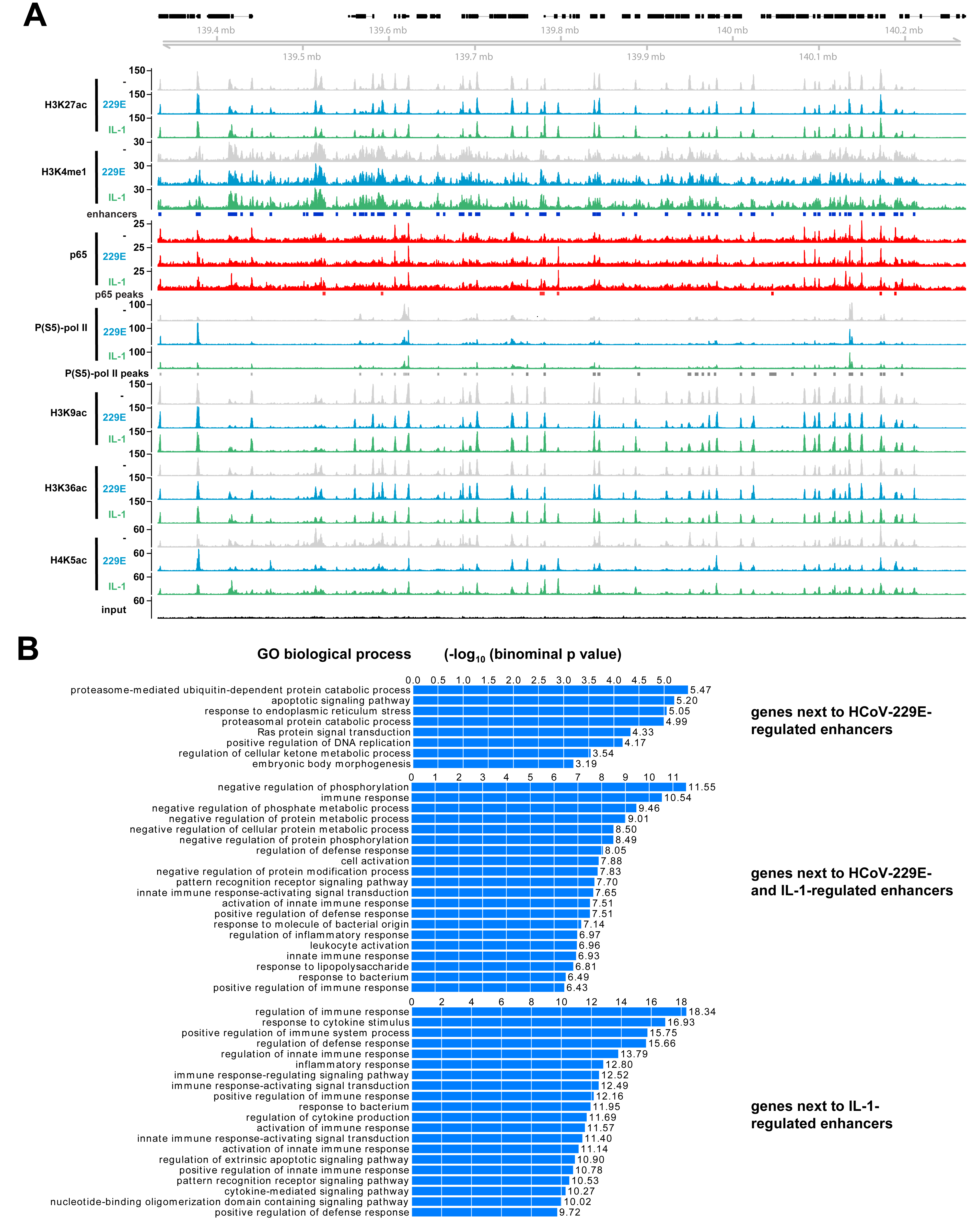

Supplement: S8 Fig — (A) Shown is an example for all ChIP-seq data obtained for HuH7 cells in this study showing non-regulated enhancers (blue bars), regions of constitutive P(S5)-pol II recruitment (gray bars), NF-κB binding (red bars) and predicted NF-κB motifs (vertical red bars). (B) Gene Ontology (GO) analyses for all annotated genes located next to the three groups of enhancers described in Fig 8C. Differentially up-regulated enhancers (as detected by > 2-fold induction of H3K27ac binding) were analyzed for over-represented GO terms amongst the genes mapped to respective enhancer intervals. Bar graphs show negative decadic logarithms of the binomial p values of significantly enriched GO terms. (TIF) [file ppat.1006286.s008.tif]

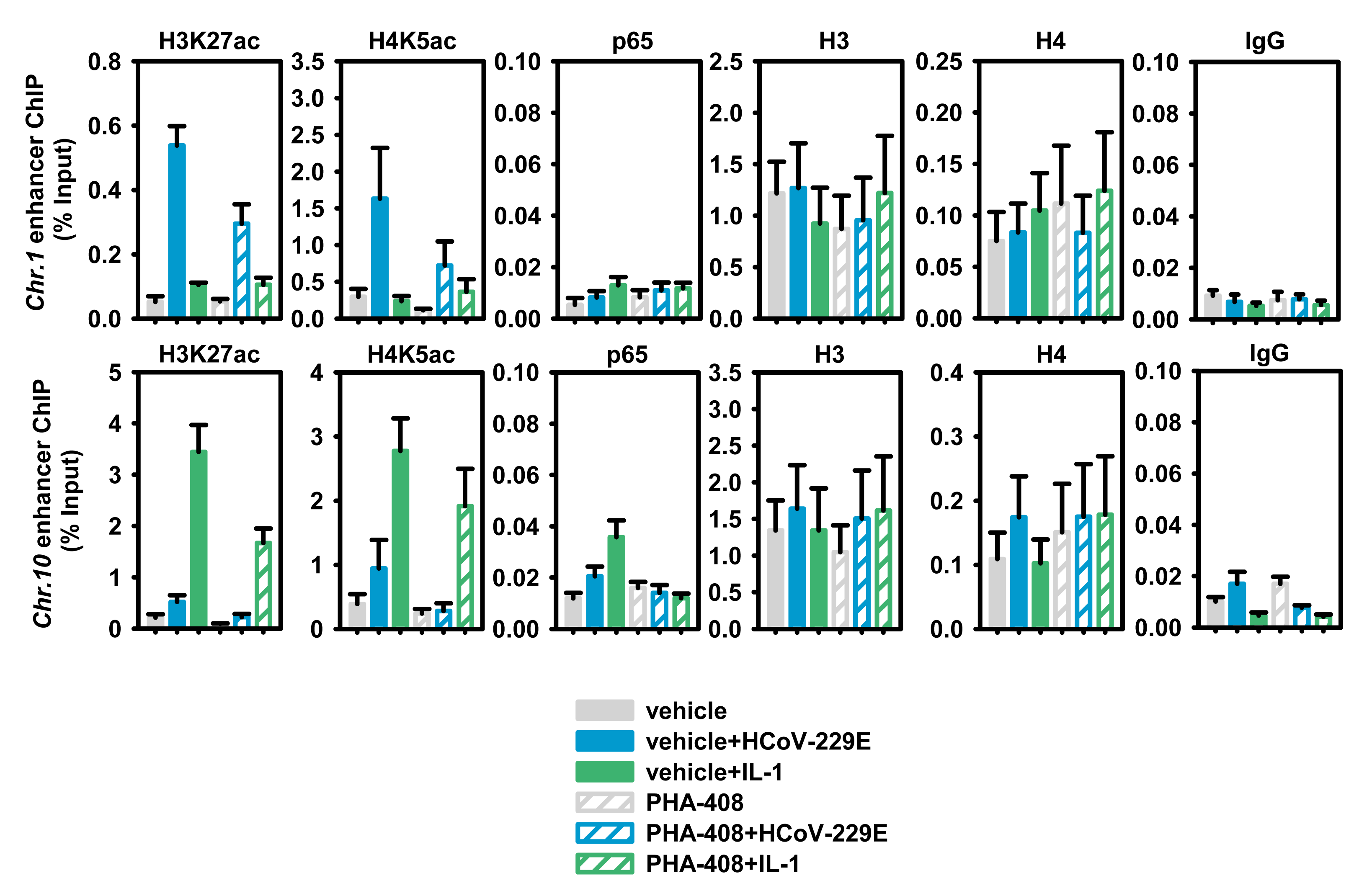

Supplement: S9 Fig — Chromatin prepared from HuH7 cells treated exactly as described in the legend of Fig 5E was used to determine by ChIP experiments the histone modifications, p65 recruitment and histone densities at the virus-specific enhancer region on Chr.1 or the IL-1-specific enhancer region on Chr. 10 shown in Fig 8D. Shown are the results from two independent ChIP-PCR experiments, IgG immunoprecipitations served as negative control. (TIF) [file ppat.1006286.s009.tif]
