# Supplementary material for: The NF-κB-dependent and -independent transcriptome and chromatin landscapes of human coronavirus 229E-infected cells
Source: PLoS Pathog. 2017 Mar 29;13(3):e1006286. doi: 10.1371/journal.ppat.1006286 (PMC5386326; doi:10.1371/journal.ppat.1006286)
Supplement: S1 Supporting Experimental Procedures — (PDF) [file ppat.1006286.s010.pdf]

S1, Supporting Experimental Procedures

Human cell lines and viruses

A549 human alveolar basal epithelial cells, HeLa cells (ATCC) and HuH7 hepatocyte derived cellular carcinoma cells (Japanese Collection of Research Bioresources (JCRB) cell bank) [1] were maintained in Dulbecco's modified Eagle's medium (DMEM), complemented with 10% fetal calf serum (FCS), 2mM L-glutamine, 100 U/ml penicillin and 100 µg/ml streptomycin. The genome sequence of the human coronavirus (strain 229E) used in this study is available from GenBank (accession number 304460). Infections of A549 and HuH7 cells were performed at 33°C using the indicated multiplicities of infection (MOI). Virus titers ($TCID_{50}$/ml) were determined using HuH7 cells. IL-1 treatment was done at 33°C using identical conditions and cell culture medium.

Materials

Human recombinant IL-1α used at 10 ng/ml in all experiments was a kind gift from Jeremy Saklatvala, Oxford, UK. The IKKβ inhibitor PHA-408 was purchased from Axon Medchem, dissolved in DMSO (1:20,000) prior to use and applied at a concentration of 5µM. Appropriate DMSO concentrations served as a vehicle control in all PHA-408 experiments. Other reagents were from Sigma-Aldrich or Thermo Fisher Scientific and were of analytical grade or better.

Antibodies against the following proteins or peptides were used: ß-actin (Calbiochem JLA20, CP01; Santa Cruz, sc-47778), eIF2α (Cell signaling #9722), P(S51)-eIF2α (Cell signaling #9721), P(S176/180)IKKα / P(S177/181)IKKβ (Cell Signaling, #2697), IKKβ (Santa Cruz (H4), sc-8014), IKKα (Santa Cruz (H-744), sc-7218), TNFAIP3 (Santa Cruz, sc-166692), p105/p50 NF-κB (Santa Cruz (E10), sc-8414), p100/p52 NF-κB (Merck Millipore 05-361), N protein (INGENASA Batch 250609), nsp8 (rabbit, gift of John Ziebuhr), P(S536)-p65 (Santa Cruz, sc-3033), p65 (Santa Cruz (C-20), sc-372), p65 (Santa Cruz (F-6), sc-8008), P(S32)-IκBa (Cell Signaling, #2859), IκBα (Cell Signaling, #9242), RNA polymerase II (Millipore, 17-620), c-Rel (Santa Cruz, sc-70), tubulin (TU-02, sc-8035). Sources of Horseradish peroxidase-coupled secondary antibodies were as follows: goat anti mouse IgG (Dako Cytomation), goat anti rabbit IgG (Pierce 31460; Lot

RB230194), TrueBlot anti mouse (#18-8817-33) or anti rabbit (#18-8816-33) IgG (Rockland).

Plasmids and transient transfections

shRNA constructs for knockdown of p65 were prepared in pSuper-Puro backbone as previously described (Handschick et al., 2014). The shRNA-encoding plasmid to knock down TNFAIP3 was obtained from Sigma (pLKO.1 shTNFAIP3 (TRCN0000050959; NM_006290). To suppress p65 or TNFAIP3, HeLa cells were transiently transfected by the calcium phosphate method. After 24 h, the medium was changed and transfected cells were selected for 48 h (for shp65) or 72 h (for shTNFAIP3) with 1 µg/ml puromycin. After selection cells were infected for another 24 h with a MOI of 1 with HCoV-229E before harvesting.

mRNA expression analysis by RT-qPCR

1-2 µg of total RNA was prepared by column purification (Macherey and Nagel or Qiagen) and transcribed into cDNA using Moloney murine leukemia virus reverse transcriptase (Fermentas) in a total volume of 20 µl. For mRNA measurement of cellular genes and HCoV-229E genomic RNA1 (nsp8) and RNA2 (Spike) as shown in Figs. 1E, 5F, 6B, 6F, S1A, S3A 2 µl of this reaction mixture were used to amplify cDNAs by RT-qPCR using Fast SYBR® Green Master Mix (ThermoFisher Scientific, 4385612) and the primers shown below. Melting curve analysis revealed a single PCR product.

cDNA	Primer se	Primer as
hDDIT3	ggaggtgcttgtgacctctg	gacctgcaagaggtcctgtc
hHerpud1	tcggaacctttcttcccctg	gctggaaccaggaaagctga
hEGR1	tctcctctcccggctcctcg	cccttttccctttctttcccctttt
hATF3	gctgcaaagtgccgaaacaa	atacacgtgggccgatgaag
hCHAC1	tggattttcgggtacggctc	tggatgctcaccatcttgtcg
hANKRD1	acgccaaagacagagaagga	ttctgccagtgtagcaccag
HCoV-229E genomic RNA 1 (nsp8)	gctgttgcaaatggttcctcac	gatgcacattcttaccatcattatcc
HCoV-229E genomic RNA 2 (Spike)	tttcaggtgatgctcacatacc	acaaactcacgaactgtcttagg

hCHUK	tcctcaagatggggagacttc	ggggacagtgaacaagtgaca
hIkBKB	cagatcatgagaaggctgacc	ggtatctaagcgcagaggca
hIkBKG	cagcatcatcgaggtcccat	ccagaaagctctggccttgta

In addition, for the experiments described in Figs. 5F, 6B, 6F, S3A, S4A and S5A, RT-qPCR reactions starting with 1 µg of total RNA were performed using assays on demand (Applied Biosystems) for *IL8* (Hs00174103_m1), *CXCL2* (Hs00236966_m1), *EGR1* (Hs00152928_m1), *IL6* (Hs00174131_m1), *DNAJB9* (Hs01573477_g1), *DDIT3* (Hs00358796_g1), *HERPUD1* (Hs01124269_m1), *TNFAIP3* (Hs00234713_m1), *ATF3* (Hs00231069_m1), *DUSP1* (Hs00610256_g1), *KLF6* (Hs00810569_m1), *NFkBIA* (Hs00153283_m1), *IkBKB* (Hs01559460_m1), *IkBKG* (Hs00415849_m1) and *GUSB* (Hs99999908_m1). All PCRs were performed as duplicate reactions on an ABI7500 real time PCR instrument. The threshold value ct for each individual PCR product was calculated by the instrument's software and ct values obtained for inflammatory mRNAs were normalized by subtracting the ct values obtained for *GUSB*. The resulting Δct values were then used to calculate relative changes of mRNA expression as ratio (R) of mRNA expression of stimulated/unstimulated or uninfected/infected cells according to the equation: $R=2^{-(\Delta ct(stimulated)-\Delta ct(unstimulated))}$.

Immunofluorescence analysis

Indirect immunofluorescence analysis was performed as described [2] using the following antibodies: N protein (INGENASA Batch 250609),
p65 (Santa Cruz, C-20, sc-372), Dylight488 donkey anti rabbit (ImmunoReagent DkxRb-003-D488NHSX, 1:200) and Cy3 donkey anti mouse (Millipore AP192C, 1:200).

Laser microdissection and single cell RT-qPCR

Cells were seeded in 30 mm circular dishes equipped with a 2 µm PEN (polyethylene naphthalate) membrane. Immunostaining and LMD were performed according to published protocols with the following modifications [3]. At the end of treatments, cells were washed with Hank's BSS (PAA Laboratories) 2 x for 5 min and fixed with 2 ml methanol for 5 min. Dishes were rinsed in Hank's BSS for 30 sec and then incubated for 3 min with 2 ml Hank's BSS/0.1% saponin plus 10% donkey serum for blocking. The membrane area was incubated with primary antibodies diluted 1:50 (N protein

INGENASA) or 1:400 P(S2)-pol II (Abcam, ab5095) in 400 µl of Hank's BSS containing 0.1% saponin (Sigma) at 37°C in a humid chamber. Immunofluorescence controls received Hank's BSS/0.1% saponin only. Thereafter, samples were washed in Hank's BSS/0.1% saponin for 30 sec. Then, the membrane area was incubated with a mixture of Cy3-conjugated (AP182C, Lot2397069, from Chemicon or Millipore; diluted 1:50) and Dylight488-conjugated (DkxMu-003-D488NHSX, Lot 18-81-101810 from Immuno reagents; diluted 1:400) antibodies in 400 µl of Hank's BSS/0.1% saponin at room temperature. Samples were washed 3 x in 2 ml Hank's BSS for 30 sec, followed by 30 sec in 2 ml DEPC H_2O and then dried for 30 min at 40°C. Then non-infected cells and virus-infected cells were detected by fluorescence microscopy, excised and collected in Eppendorf tubes using a Leica LMD6000 system. For microarray analysis (Fig. 1B-D), at least 5,000 cells were extracted, pooled and RNA was isolated using the Arcturus® PicoPure® RNA Isolation Kit (Cat no.: 12204-01; Lot: 1402093) according to the manufacturer's protocol. RNA quality and concentration were measured with a RNA PicoChip (Lot PB05BK30) on an Agilent 2100 bioanalyzer according to the manufacturer's protocol. For mRNA expression analysis by RT-qPCR (Fig. 1E), 400 pg of total RNA was reverse transcribed and amplified using the TaqMan® PreAmp Cells-to-CT™ Kit (ABI). After cDNA synthesis, gene-specific primers were used for 15 cycles of pre-amplification and for PCR reactions according to the manufacturer's instructions. PCR products were detected by TaqMan® probes as described above. Taqman probes and primer pairs detecting *GUSB* were used for normalization.

Cell lysis

For whole cell extracts (Fig. 4A) cells were lysed in Triton cell lysis buffer (10mM Tris, pH 7.05, 30mM $NaPP_i$, 50mM NaCl, 1% Triton X-100, 2mM Na_3VO_4, 50mM NaF, 20mM ß-glycerophosphate and freshly added 0.5mM PMSF, 2.5µg/ml leupeptin, 1.0 µg/ml pepstatin, 1µM microcystin) as previously described [4]. Cytosolic, soluble and chromatin-associated nuclear fractions (Fig. 4B) were prepared as described in [5]. For separation of soluble and insoluble nuclear fractions, cells were lysed in lysis buffer 1 (20mM HEPES (pH 8.0), 10mM KCl, 1mM $MgCl_2$, 0.1% Triton X-100, 20% Glycerol, 50mM NaF, Roche protease inhibitor mix, 1µM microcystin, 1mM Na_3VO_4) for 10min on ice and centrifuged for 1min at 2,300xg at 4°C. The supernatant (C, cytosol) was

removed and the pellet was lysed in lysis buffer 2 (20mM HEPES (pH 8.0), 2mM EDTA, 400mM NaCl, 0.1% Triton X-100, 20% Glycerol, 50mM NaF, Roche protease inhibitor mix, 1μM microcystin, 1mM Na_3VO_4) for 20min on ice, vortexed twice, and centrifuged at 18,000 x g at 4°C for 5min. The supernatant (N1, soluble nuclear fraction) was removed and the pellet was lysed in 20mM Tris (pH 7,5), 2mM EDTA, 150mM NaCl, 1% SDS, 1% NP-40, 50mM NaF, Roche protease inhibitor mix, 1μM microcystin, 1mM Na_3VO_4 for 50min on ice, repeatedly vortexed and centrifuged at 18,000 x g at 4°C for 5min. Then, the supernatant (N2, insoluble nuclear fraction) was collected. Cell lysates or subcellular fractions were subjected to SDS-PAGE on 7-12.5% gels and immunoblotting was performed as described below.

Immunoblotting

Immunoblotting was performed essentially as described (Hoffmann et al., 2005). Proteins were separated on SDS-PAGE and electrophoretically transferred to PVDF membranes (Roth, Roti-PVDF (0,45μm)). After blocking with 5% dried milk in Tris-HCl-buffered saline/0.05% Tween (TBST) for 1 h, membranes were incubated for 12-24 h with primary antibodies, washed in TBST and incubated for 1-2 h with the peroxidase-coupled secondary antibody. Proteins were detected by using enhanced chemiluminescence (ECL) systems from Millipore or GE Healthcare. Images were acquired and quantified using a Kodak Image Station 440 CF and the software Kodak 1D, 3.6, or the ChemiDoc Touch Imaging System (BioRad) and the software ImageLab V_5.2.1 (Bio-Rad).

Microarray experiments

Purified total RNA was amplified and Cy3-labeled using the LIRAK kit (Agilent) following the kit instructions. Per reaction, 200 ng of total RNA was used. The Cy3-labeled aRNA (amplified antisense RNA) was hybridized overnight to 8x60K 60mer oligonucleotide spotted microarray slides (Agilent Technologies, design ID 039494). Hybridization and subsequent washing and drying of the slides were performed following the Agilent hybridization protocol. The dried slides were scanned at 2 μm/pixel resolution using the InnoScan is900 (Innopsys, Carbonne, France). Image analysis was performed with Mapix 6.5.0 software, and calculated values for all spots were saved as GenePix results files. Stored data were evaluated using the R software and the limma package from BioConductor [6-8]. Log_2 mean spot signals were taken for further

analysis. Data were background corrected using the NormExp procedure on the negative control spots and quantile-normalized before averaging. For filtering of “expressed” genes the distribution of log_2 signal intensities of the negative control spots was determined for each array. A regular spot was considered “present” when its signal was 3 times higher than the interquartile width of the 3rd quartile. A gene was considered “expressed” on an array when at least 50% of the spots for this gene were “present”. A gene was considered “expressed” in a group of biological samples, when this gene was judged “expressed” in at least 50% of the arrays of this group. For Fig. 1A, selections of regulated genes from single hybridizations were made based on the noise observed between hybridizations of heat-inactivated controls. A heuristic noise-band function was set up to distinguish a regulation from noise so that the probability of a falsely identified regulated gene is < 0.1%. The noise-band function is $\pm 1.5 \cdot 8/A$, where A is the average log_2 signal. KEGG pathway analyses were done using gene set tests on the ranks of the t-values (when available) or log_2 fold-changes [6, 9]. The significances for the KEGG pathway perturbations were calculated by the function geneSetTest from limma. The statistics were calculated for all three alternatives “mixed”, “up”, and “down”, and the smallest p value was taken and Bonferroni corrected. In this analysis only genes were considered with average signal intensities in at least one group that were considerably higher than the average signal of negative controls on the arrays as described above. The significances for the over-representation of selected gene lists were calculated by Fisher’s exact test. As for the gene set tests, only “expressed” genes were considered.

Chromatin immunoprecipitation

One 145-cm^2 dish of confluent HuH7 or HeLa cells (corresponding to approximately 1.0 $x10^7$ cells), treated as described in the figure legends, was used for each condition. Cross-linking was performed *in vivo* with 1% formaldehyde added directly to the medium followed by 10 min incubation time at room temperature. After stopping the cross-linking reaction by adding 0.1 M glycine for 5 min the cells were collected by scraping and centrifugation at 1,610 x g (5 min, 4°C), washed in cold PBS containing 1 mM PMSF and centrifuged again at 1,610 x g (5 min, 4°C). Cells were lysed for 10 min on ice in 1 ml ChIP lysis buffer (1% SDS, 10 mM EDTA, 50 mM Tris pH 8.1, 1 mM PMSF, Roche protease inhibitor mix). The DNA was sheared by sonication (28 x 30 s on / 30 s off;

Bioruptor, Diagenode) and lysates were cleared by centrifugation at 16,100 x g at 4°C for 15 min. Supernatants were collected and stored in aliquots at –80°C for subsequent ChIP. For determination of DNA concentration, 20 µl of sheared lysate was diluted with 100 µl TE buffer including 10 µg/ml RNAse A. After 30 min at 37°C, 3.8 µl proteinase K (20mg/ml) and 1% SDS was added and incubated for at least 2 h at 37°C followed by overnight incubation at 65°C. Samples were resuspended in two volumes of buffer NTB (Macherey & Nagel) and DNA was purified using Nucleo Spin columns (Macherey & Nagel) according to the manufacturer's instructions. DNA was eluted with 50 µl 5 mM Tris pH 8.5 and concentration was determined by Nano Drop. For ChIP, 1-3 µg of the above indicated antibodies were used. Antibodies were added to precleared lysate volumes equivalent to 20 µg of chromatin. Then, 900 µl of ChIP dilution buffer (0.01% SDS, 1.1% Triton X-100, 1.2 mM EDTA, 167 mM NaCl, 16.7 mM Tris/HCl pH 8.1) were added and the samples were rotated at 4°C overnight. Thereafter, 30 µl of a protein A/G sepharose mixture, pre-equilibrated in ChIP dilution buffer was added to the lysates and incubation continued for 2 h at 4°C. Beads were collected by centrifugation, washed once in 900 µl ChIP low salt buffer (0.1% SDS, 1% Triton X-100, 2mM EDTA, 20 mM Tris pH 8.1, 150 mM NaCl), once in 900 µl ChIP high salt buffer (0.1% SDS, 1% Triton X-100, 2 mM EDTA, 20 mM Tris pH 8.1, 500 mM NaCl), once in 900 µl ChIP LiCl buffer (0.25M LiCl, 1% NP40, 1% desoxycholate, 1 mM EDTA, 10 mM Tris pH 8.1) and twice in 900 µl ChIP TE buffer (10 mM Tris pH 8.1, 1 mM EDTA) for 5 min at 4°C. Beads were finally resuspended in 100 µl TE buffer including RNAse A (10 mg/ml). In parallel, 1/10 volume of the initial lysate (input samples) was diluted with 100 µl TE buffer including 10 µg/ml RNAse A. After 30 min at 37°C, 3.8 µl proteinase K (20 mg/ml) and 1% SDS were added and both, input and immunoprecipitates were incubated for at least 2 h at 37°C followed by overnight incubation at 65°C. Samples were resuspended in two volumes of buffer NTB (Macherey & Nagel) and DNA was purified using Nucleo Spin columns (Macherey & Nagel) according to the manufacturer's instructions. DNA was eluted with 50 µl 5 mM Tris pH 8.5 and stored at –20°C until further use. ChIP antibodies used in this study: normal rabbit IgG (Cell Signaling, #2729), p65 (Santa Cruz, C-20, sc-372), pol II (Millipore, 17-620), P(S5)-pol II (Abcam, ab5131), H3 (Abcam, ab1791), H3K9ac (Millipore, 07-352), H3K27ac (Diagenode, 174-

050), H3K4me1 (Abcam, ab8895), H4K5ac (Millipore, 17-10045), H4 (Millipore, 17-10047), CoV N protein (Ingenasa, M.30.Hco.I1E7), H3K36ac (Diagenode, C154 10307).

Quantification of ChIP DNA by real-time PCR

PCR products derived from ChIP were quantified by real time PCR using the Fast ABI 7500 instrument (Applied Biosystems). The reaction mixture contained 2 µl of ChIP or input DNA (diluted 1:10 to represent 1% of input DNA), 0.25 µM of primers and 10 µl of Fast Sybr Green Mastermix (2x) (Applied Biosystems) in a total volume of 20 µl. PCR cycles were as follows: 95°C (20 s), 40x (95°C (3 s), 60°C (30 s)). Melting curve analysis revealed a single PCR product. Calculation of enrichment by immunoprecipitation relative to the signals obtained for 1% input DNA was performed according to the following equation: percent of (input)=$2^{-(Ct\ sample-Ct\ input)}$. ChIP-PCR primers were as follows:

Locus	Primer se	Primer as
IL8 promoter	aagaaaactttcgtcatactccg	tggctttttatatcatcaccctac
CHAC1 promoter	gaagttgattggccaaaagg	gctgcagactcctgcttcat
ANKRD1 promoter	agctgtcccctgactcttga	caacctgggaaccgaagtaa
CXCL2 promoter	gtcagacccggacgtcact	accccttttatgcatggttg
Enhancer Chr1	gaccagattccagtcgaagg	gtaaccctgccagaaggtga
Enhancer Chr10	cagacaaggctcactgcttg	cactgctgaggacccctaga

ChIP-seq analyses and data visualization

Deep sequencing of ChIP DNA

Samples were prepared as described for ChIP, but the elution step after DNA purification was performed with H_2O instead of elution buffer. If necessary samples generated with the same antibody were pooled and volume was reduced by evaporation to 30 µl to obtain at least 10 ng of total DNA. Sequencing libraries were prepared from 10 ng of immunoprecipitated DNA with the NEBNext ChIP-Seq Library Prep Reagent (New England Biolabs) according to manufacturer's instructions. Cluster generation was performed using the cBot (Illumina Inc.). Sequencing was done on the HiSeq 2500 (Illumina Inc.) using TruSeq SBS Kit v3 - HS (Illumina) for 50 cycles. Image analysis and base calling were performed using the Illumina pipeline v 1.8 (Illumina Inc.).

Processing of ChIP-seq reads

ChIP-Seq reads were converted to fastq format and aligned to a precompiled hg19 reference index with BOWTIE with *-k* option set to 1 [10, 11]. Sequencing data were controlled for general quality features using FastQC (http://www.bioinformatics.babraham.ac.uk/projects/fastqc/). Unambiguously mapped and unique reads were kept for subsequent generation of binding profiles and calling of peaks using MACS v1.41 [12] and PeakRanger [13] at default settings. All downstream analyses were done in R/BioConductor (http://www.bioconductor.org) [7].

Peak analysis

Peaks identified by MACS or PeakRanger at a Poisson p value < 10^{-5} and an FDR < 5% were used for intersection analysis to determine the overlap in pairwise comparisons. Two peaks were determined to be overlapping in case they had a minimal overlapping interval of 1 bp. In order to identify differentially bound regions we collapsed all binding regions determined for a given factor under different conditions and extracted the read numbers mapping to these collapsed intervals. DESeq [14] was used to normalize between samples and in order to determine changes in occupancy and genes were ranked accordingly. In case we were studying the overlap of multiple binding factors at once we used the *multiinter* function of the BedTools suite [15] with the -cluster option turned on. All intervals like enhancers or transcription binding peaks were annotated with respect to human hg19 RefSeq annotations downloaded from Illumina's iGenome repository. In order to assign genes to peaks we linked each peak with the next gene (maximum distance between peak border and transcriptional start site was set to 500 kb). Gene ontology analysis of differentially bound enhancers was done with GREAT using default settings [16].

Visualization of binding profiles

After extension of reads continuous coverage vectors were calculated and normalized per million reads to account for differential library sizes. These data were used to collect data in windows of different sizes spanning features of interest (e.g. transcription factor peaks). The binding data was binned across binding sites in 50 bp windows and the mean was calculated at each position in order to generate cumulative average binding profiles.

For representation in genome browsers, profiles were additionally smoothed using kernel regression estimates. Data was visualized using the Gviz BioConductor package.

Enhancer analysis

The principal set of enhancers in this study was determined as the collapsed overlap of H3K4me1 and H3K27ac peaks as determined in all of the 3 experimental conditions (control, IL-1, HCoV-229E). IL-1- and CoV-dependent enhancers were defined as the subset of these enhancers marked by substantial induction of H3K27ac (> 2-fold) when comparing IL-1 treatment or HCoV-229E infection with the control treatment of the cells.

Motif search

We extracted the sequences in a +/- 50bp window around the peak maxima and discrete sub-peak maxima (called by the PeakSplitter application: http://www.ebi.ac.uk/research/bertone/software) from top ranked peaks. These sequences were used to run MEME-ChIP (http://meme.ebi.edu.au/) in order to identify *de novo* motifs and known motifs enriched within peak regions of respective factors [17]. In order to identify motifs within regulated enhancers with higher fidelity, we pursued an alternative strategy. Within enhancers we found that in many cases p65 binding sites were found at local minima of corresponding histone modification ChIP-seq data. In order to better define potential transcription factor binding motif containing sequences we implemented a tool to identify "valleys" within the coverage profiles for H3K27ac data. Therefore, we calculated a "valley score" across the analyzed enhancer intervals at 10 bp resolution similar to what has been described in a previous study [18]. Bins assigned as valleys were stitched together to larger regions in case they occured within 50 bp. Identified valley intervals were validated by optical inspection of profiles in the genome browser and by plotting the average H3K27ac coverage across all identified enhancer valleys. Instead of the complete enhancer sequence we submitted only the sequences corresponding to valley regions to MEME-ChIP, thereby excluding more than 80 % of the original enhancer sequences.

Statistics

Statistics (Mann-Whitney Rank, Wilcoxon signed rank or t-tests) were calculated using R, SigmaPlot11, GraphPadPrism6.0 and MS EXCEL2010.

Supplemental references

1. Nakabayashi H, Taketa K, Miyano K, Yamane T, Sato J. Growth of human hepatoma cells lines with differentiated functions in chemically defined medium. Cancer Res. 1982;42(9):3858-63. PubMed PMID: 6286115.
2. Handschick K, Beuerlein K, Jurida L, Bartkuhn M, Muller H, Soelch J, et al. Cyclin-dependent kinase 6 is a chromatin-bound cofactor for NF-kappaB-dependent gene expression. Mol Cell. 2014;53(2):193-208. doi: 10.1016/j.molcel.2013.12.002. PubMed PMID: 24389100.
3. Fend F, Emmert-Buck MR, Chuaqui R, Cole K, Lee J, Liotta LA, et al. Immuno-LCM: laser capture microdissection of immunostained frozen sections for mRNA analysis. Am J Pathol. 1999;154(1):61-6. doi: 10.1016/S0002-9440(10)65251-0. PubMed PMID: 9916919; PubMed Central PMCID: PMC1853427.
4. Jurida L, Soelch J, Bartkuhn M, Handschick K, Muller H, Newel D, et al. The Activation of IL-1-Induced Enhancers Depends on TAK1 Kinase Activity and NF-kappaB p65. Cell Rep. 2015;10:726-39.
5. Handschick K, Beuerlein K, Jurida L, Bartkuhn M, Muller H, Soelch J, et al. Cyclin-dependent kinase 6 is a chromatin-bound cofactor for NF-kappaB-dependent gene expression. MolCell. 2014;53(2):193-208.
6. Ritchie ME, Phipson B, Wu D, Hu Y, Law CW, Shi W, et al. limma powers differential expression analyses for RNA-sequencing and microarray studies. Nucleic Acids Res. 2015;43(7):e47. doi: 10.1093/nar/gkv007. PubMed PMID: 25605792; PubMed Central PMCID: PMCPMC4402510.
7. Gentleman RC, Carey VJ, Bates DM, Bolstad B, Dettling M, Dudoit S, et al. Bioconductor: open software development for computational biology and bioinformatics. Genome Biol. 2004;5(10):R80. doi: 10.1186/gb-2004-5-10-r80. PubMed PMID: 15461798; PubMed Central PMCID: PMC545600.
8. RDevelopmentCoreTeam. R: A language and environment for statistical computing. R Foundation for Statistical Computing, Vienna, Austria. ISBN 3-900051-07-0, URL http://www.R-project.org. 2016.
9. Silver JD, Ritchie ME, Smyth GK. Microarray background correction: maximum likelihood estimation for the normal-exponential convolution. Biostatistics. 2009;10(2):352-63. doi: 10.1093/biostatistics/kxn042. PubMed PMID: 19068485; PubMed Central PMCID: PMCPMC2648902.
10. Langmead B. Aligning short sequencing reads with Bowtie. Current protocols in bioinformatics / editoral board, Andreas D Baxevanis [et al]. 2010;Chapter 11:Unit 11 7. doi: 10.1002/0471250953.bi1107s32. PubMed PMID: 21154709; PubMed Central PMCID: PMC3010897.
11. Langmead B, Trapnell C, Pop M, Salzberg SL. Ultrafast and memory-efficient alignment of short DNA sequences to the human genome. Genome Biol. 2009;10(3):R25. Epub 2009/03/06. doi: gb-2009-10-3-r25 [pii]10.1186/gb-2009-10-3-r25. PubMed PMID: 19261174; PubMed Central PMCID: PMC2690996.
12. Zhang Y, Liu T, Meyer CA, Eeckhoute J, Johnson DS, Bernstein BE, et al. Model-based analysis of ChIP-Seq (MACS). Genome Biol. 2008;9(9):R137. Epub

2008/09/19. doi: gb-2008-9-9-r137 [pii]10.1186/gb-2008-9-9-r137. PubMed PMID: 18798982; PubMed Central PMCID: PMC2592715.

13. Feng X, Grossman R, Stein L. PeakRanger: a cloud-enabled peak caller for ChIP-seq data. BMC Bioinformatics. 2011;12:139. Epub 2011/05/11. doi: 1471-2105-12-139 [pii]10.1186/1471-2105-12-139. PubMed PMID: 21554709; PubMed Central PMCID: PMC3103446.
14. Anders S, Huber W. Differential expression analysis for sequence count data. Genome Biol. 2010;11(10):R106. doi: 10.1186/gb-2010-11-10-r106. PubMed PMID: 20979621; PubMed Central PMCID: PMC3218662.
15. Quinlan AR, Hall IM. BEDTools: a flexible suite of utilities for comparing genomic features. Bioinformatics. 2010;26(6):841-2. doi: 10.1093/bioinformatics/btq033. PubMed PMID: 20110278; PubMed Central PMCID: PMC2832824.
16. McLean CY, Bristor D, Hiller M, Clarke SL, Schaar BT, Lowe CB, et al. GREAT improves functional interpretation of cis-regulatory regions. Nature biotechnology. 2010;28(5):495-501. doi: 10.1038/nbt.1630. PubMed PMID: 20436461.
17. Machanick P, Bailey TL. MEME-ChIP: motif analysis of large DNA datasets. Bioinformatics. 2011;27(12):1696-7. Epub 2011/04/14. doi: btr189 [pii]10.1093/bioinformatics/btr189. PubMed PMID: 21486936; PubMed Central PMCID: PMC3106185.
18. Ramsey SA, Knijnenburg TA, Kennedy KA, Zak DE, Gilchrist M, Gold ES, et al. Genome-wide histone acetylation data improve prediction of mammalian transcription factor binding sites. Bioinformatics. 2010;26(17):2071-5. doi: 10.1093/bioinformatics/btq405. PubMed PMID: 20663846; PubMed Central PMCID: PMC2922897.
